# A single-cell atlas of *Plasmodium falciparum* transmission through the mosquito

**DOI:** 10.1038/s41467-021-23434-z

**Published:** 2021-05-27

**Authors:** Eliana Real, Virginia M. Howick, Farah A. Dahalan, Kathrin Witmer, Juliana Cudini, Clare Andradi-Brown, Joshua Blight, Mira S. Davidson, Sunil Kumar Dogga, Adam J. Reid, Jake Baum, Mara K. N. Lawniczak

**Affiliations:** 1 Department of Life Sciences, Imperial College London, London, UK; 2 Parasites and Microbes Programme, Wellcome Sanger Institute, Hinxton, UK; 3 Institute of Biodiversity, Animal Health and Comparative Medicine, College of Medical Veterinary and Life Sciences, University of Glasgow, Glasgow, UK; 4 Wellcome Centre for Integrative Parasitology, College of Medical Veterinary and Life Sciences, University of Glasgow, Glasgow, UK; 5 Department of Infectious Disease, Imperial College London, London, UK

**Keywords:** RNA sequencing, Parasite biology, Malaria

## Abstract

Malaria parasites have a complex life cycle featuring diverse developmental strategies, each uniquely adapted to navigate specific host environments. Here we use single-cell transcriptomics to illuminate gene usage across the transmission cycle of the most virulent agent of human malaria - *Plasmodium falciparum*. We reveal developmental trajectories associated with the colonization of the mosquito midgut and salivary glands and elucidate the transcriptional signatures of each transmissible stage. Additionally, we identify both conserved and non-conserved gene usage between human and rodent parasites, which point to both essential mechanisms in malaria transmission and species-specific adaptations potentially linked to host tropism. Together, the data presented here, which are made freely available via an interactive website, provide a fine-grained atlas that enables intensive investigation of the *P. falciparum* transcriptional journey. As well as providing insights into gene function across the transmission cycle, the atlas opens the door for identification of drug and vaccine targets to stop malaria transmission and thereby prevent disease.

## Abstract (web summary)

Here the authors use single-cell RNA-seq to profile the transmission stages of the human malaria parasite *Plasmodium falciparum* as it progresses through the *Anopheles* mosquito. They highlight unique patterns of gene usage throughout this development and identify potential pleiotropic genes that function at multiple life cycle stages.

## Introduction

Malaria parasite transmission to and from the mosquito vector is accompanied by a precipitous drop in parasite population numbers^1,2^. The bottleneck that transmission affords has, therefore, long been considered an attractive target for antimalarial or vaccine interventions. In a highly orchestrated series of developmental transitions taking place inside the mosquito, parasites taken up during a blood meal complete sexual development, fertilization, and recombination; differentiate into invasive ookinetes that colonize the midgut; develop into oocysts where population expansion occurs; and emerge as invasive sporozoites that fill the mosquito salivary glands and can be transmitted to a new host during a mosquito bite^3^. After a relatively brief stint in the skin, some injected sporozoites will reach the liver, where they undergo a phase of profound population expansion, followed by a release of merozoites that enter the bloodstream^4^. This heralds the beginning of the cyclical asexual cycle, where merozoites invade erythrocytes and develop intra-erythrocytically, further expanding parasite numbers, with some parasites also developing into sexual, transmissible forms that can perpetuate the transmission cycle when taken up in the blood meal of a mosquito.

Single-cell RNA sequencing (scRNA-seq) has transformed our ability to resolve cell-level heterogeneity in complex cell populations. Recent scRNA-seq views across the full life cycle of *Plasmodium* parasites, the etiological agents of malaria, have captured fine-scale developmental transitions driving progression through the life cycle at high resolution^5–9^. The Malaria Cell Atlas^6,8^ was established as a data resource and website to provide an accessible route for scientists to explore patterns of gene expression in individual parasites across multiple developmental stages and parasite species. However, its application to *P. falciparum*, the species responsible for the vast majority of human death and disease has been thus far limited to the blood stages. Here we add a chapter to the atlas, completing a scRNA-seq survey of the transmission stages of *P. falciparum* from the sexual forms transmitted to the *Anopheles* mosquito to the invasive sporozoite delivered to the human host. Understanding these key developmental transitions in parasite biology is essential in order to devise innovative strategies for tackling malaria, a task rendered more urgent by the ongoing spread of antimalarial drug resistance and lack of a vaccine that affords long-term protection.

## Results

### Single-cell transcriptomics informs patterns of gene usage across the life cycle

**Fig. 1:**
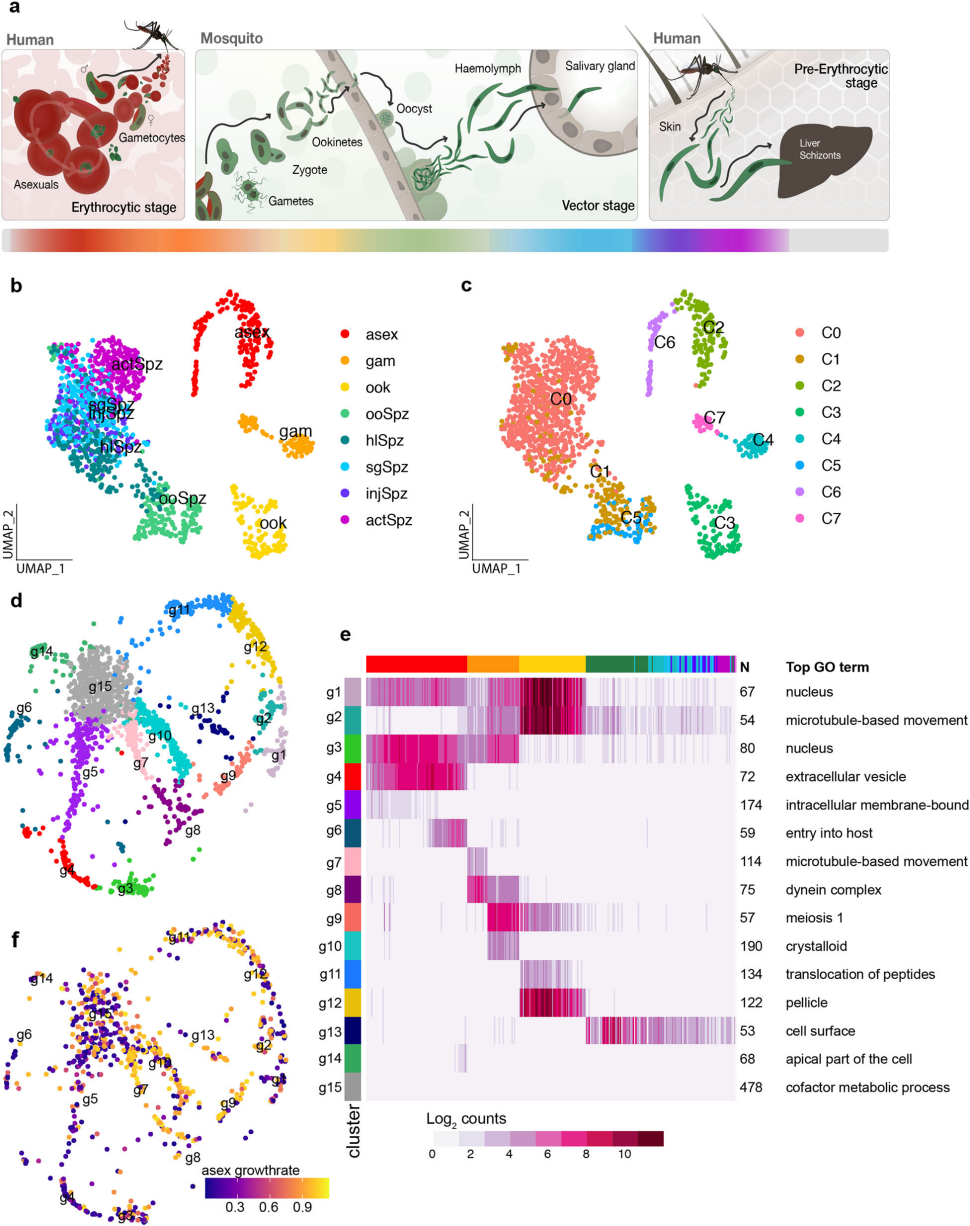
A single-cell transcriptome atlas of *P. falciparum* transmission. **a** The life cycle of *P. falciparum* includes asexual replication and sexual development in red blood cells (erythrocytic stages). Sexual stage gametocytes are then taken up in the mosquito blood meal, where they undergo fertilization and develop into the ookinete form that crosses the mosquito midgut epithelium and begins sporogonic development along the basal lamina as an oocyst. Sporozoites are released from mature oocysts to the hemolymph where they travel to and invade the salivary glands. A subset of these sporozoites may be injected into the next human host where they migrate through the dermis on their way to the liver (pre-erythrocytic stages). The color bar below represents the different life cycle stages reflected in (**b**). **b** UMAP of single-cell transcriptomes from across asexual and transmission stages of the parasite. Stage abbreviations: asex (asexual blood stages), gam (male and female gametocytes), ook (ookinete), ooSpz (oocyst sporozoites), hlSpz (hemolymph sporozoites), sgSpz (salivary gland sporozoites), injSpz (sporozoites released by mosquito bite), actSpz (activated sporozoites). Asexual stage transcriptomes have been previously published^6^. **c** Louvain clustering of single-cell transcriptomes identified eight distinct clusters. In order to get a more equal distribution of numbers of cells across clusters, clusters C0 and C1 were downsampled to 100 cells each prior to making the gene graph (**d**–**f**). **d** A k-nearest neighbors (kNN) force-directed graph of 1797 highly variable genes across cell types. Each node represents a gene. Nodes are colored by their graph-based spectral clustering assignment (g1–g15). **e** A heatmap of mean expression of the genes in each cluster across 623 subsampled cells. Cells are ordered by their developmental progression along the life cycle. Cells from asexual, ookinete, and sporozoite stages were ordered according to their computed pseudotime values (Supplementary Figs. 5 and 6). Gametocytes are grouped based on cluster assignment from (**c**), where C7 are male gametocytes and C4 are female/early gametocytes (see Supplementary Fig. 2 for more detailed analysis). The male gametocyte cells are ordered before the female gametocytes in the heatmap. Stages are represented by the colors in (**a**, **b**). The number of genes (*N*) and the top-enriched GO term (Top GO term, FET one-sided) in each cluster is indicated next to the corresponding row. Bonferroni-adjusted *p* values for this analysis are shown in Supplementary Data 4. **f** The gene graph colored by the relative growth rate of orthologous knockout mutants in *P. berghei* asexual blood-stage parasites^15^.

To capture human malaria transmission at single-cell resolution, we assembled a comprehensive scRNA-seq data set across key stages of the *P. falciparum* life cycle (Fig. 1a). Distinct morphological forms of the parasite were purified based either on the expression of known stage-specific markers (circumsporozoite protein (CSP) and ookinete surface protein P25 (Pfs25) for sporozoites and ookinetes, respectively) or the RNA and DNA content (stage V gametocytes) (Supplementary Figs. 1–3). Poor quality cells were excluded per stage based on the distribution of the number of reads and genes detected per cell (Supplementary Fig. 4; Supplementary Table 1; Supplementary Data 1–3). As observed previously^8^, the number of genes detected was dependent on parasite stage, ranging from 119 in sporozoites to 1497 in gametocytes. This is consistent with smaller cells having less total mRNA^10^, and likely reflects biological differences in the mRNA content of each cell type^10^. After quality control, our data set comprised 1467 single-cell transcriptomes, of 1808 originally sequenced, spanning the broad developmental transitions that underpin host-to-vector and vector-to-host transmission. We next integrated this transmission data set with our published collection of 161 single-cell transcriptomes of mixed *P. falciparum* asexual stages^6^, which enabled us to reconstitute the *P. falciparum* life cycle almost entirely, with the exception of exo-erythrocytic schizogony (liver stages). In order to visualize *P. falciparum* single cells across the life cycle, we performed dimensionality reduction using UMAP and clustered cell transcriptomes using Louvain clustering^11–13^. As expected, cells from the same stage grouped together in two-dimensional space (Fig. 1b) and formed distinct cell clusters (Fig. 1c; Supplementary Data 2). We were also able to observe clear cellular trajectories that are likely to reflect within-stage developmental paths (Fig. 1b, c; Supplementary Data 2). For example, the distribution of asexual parasites in a two-dimensional space clearly shows developmental progression along the intraerythrocytic developmental cycle (IDC) (Supplementary Fig. 5)^6^. Likewise, the trajectory displayed by ookinete cells is consistent with a range of developmentally intermediate states along the known ookinete maturation path (Supplementary Fig. 6)^14^.

To globally understand patterns of gene co-expression across the life cycle, we built a k-nearest neighbors (kNN) graph using the top highly variable genes (HVGs) in the data set. Each gene was assigned to one of 15 co-expression modules using graph spectral clustering (denoted with g, Fig. 1d; Supplementary Data 1), allowing us to predict gene function using a “guilt-by association” approach. The majority of gene clusters showed stage-specific expression patterns and were enriched for GO terms expected for each stage (Fig. 1e; Supplementary Fig. 7; Supplementary Data 4). To further validate the predictive value of this approach, we overlaid our gene graph with data from two genome-wide essentiality screens in *Plasmodium* and found that clusters enriched for dispensable genes (normal-growth rate in ref. ^15^ and mutable in ref. ^16^) were highly expressed in transmission stages and those enriched for essential genes were highly expressed in asexual blood stages (Fig. 1e, f; Supplementary Fig. 8; Supplementary Table 2). Taken together, this links gene usage across the life cycle to stage-specific functions allowing us to predict functionality based on co-expression patterns.

Other gene clusters were more ubiquitously expressed, possibly reflecting shared functional strategies across the life cycle. For instance, cluster g1, which features two putative readers of post-translational histone modifications, namely bromodomain protein 1 (BDP1) and the EELM2-domain-containing PF3D7_0519800, contains genes that tend to be expressed throughout the IDC and sexual stages, where epigenetic control of developmental processes has long been recognized^17,18^. Here, we find that these genes are also expressed in ookinetes, implying that epigenetic mechanisms may be more widely used across the life cycle^19^. Both BDP1 and EELM2 proteins have known or predicted links to apicomplexan AP2 transcription factors (ApiAP2 TFs)^20–22^. Significantly, two ApiAP2 TFs for which interactions with protein complexes containing BDP1 or EELM2 have been experimentally demonstrated are also expressed in this cluster (PfAP2Tel/SP3 and PF3D7_1107800, respectively)^21,22^. In addition, BDP1 has been shown to associate with the promoter regions of seven ApiAP2 TFs^20^ whose expression we find to be correlated with gene clusters associated with asexual development and host-to-vector transmission (Supplementary Fig. 9; see Supplementary Data 5 for an analysis of enriched DNA motifs in each cluster), thus expanding the reach of this gene regulatory network. A total of 18 genes without predicted functions are co-transcribed in cluster g1 and as such could represent additional links between chromatin remodeling/targeting and AP2-dependent gene expression (Supplementary Fig. 10; Supplementary Data 1).

The majority of genes in the *P. falciparum* genome have one-to-one orthologs with *P. berghei* (80%). We, therefore, explored whether any clusters were enriched for genes that lack orthologs between the two species. This analysis revealed that every cluster had some clade-specific genes present, possibly reflecting species-specific adaptive mechanisms present across the life cycle (Supplementary Figs. 11a, b, and 12). Cluster g15, which is the largest gene cluster by more than twofold showed low levels of expression across the life cycle and had the highest proportion of genes lacking orthologs, with a large number of *P. falciparum* variant multigene families (e.g., stevors, rifs) associated with host interactions in blood stages (GO:0018995, Bonferroni-adjusted *p* value = 3.58 × 10^−9^)^23^. Clusters associated with mosquito stages and vector-to-host transmission (g1, g2, g9, g12, g13), in contrast, had proportionally more genes with *P. berghei* orthologs. Despite being enriched for one-to-one orthologs, two of these clusters (g2, g13) showed the lowest amino-acid conservation scores across the gene graph (Supplementary Fig. 11c, d; Supplementary Table 2). In addition, g13, which features genes encoding surface proteins with essential roles in transmission (Supplementary Data 4), had the highest median global differentiation score^24^ (Supplementary Fig. 11e). This suggests that protein interactions that underpin transmission might have been shaped by coevolution of parasite and host and may account, at least in part, for host specificity/tropism in *Plasmodium* infections.

### Integration across species reveals conserved and divergent patterns of gene expression

**Fig. 2:**
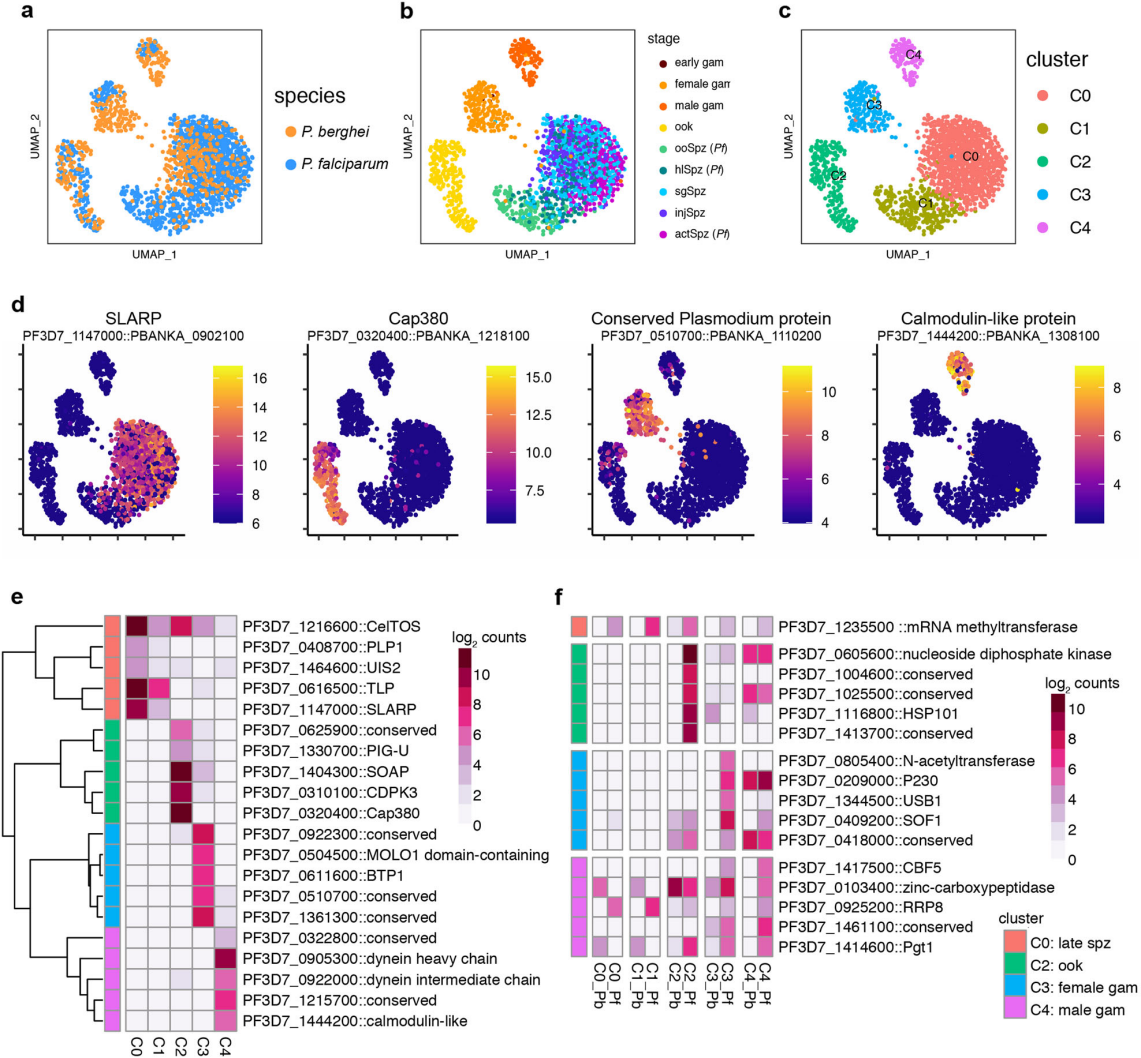
Integration of *P. falciparum* and *P. berghei* transmission data sets. To identify conserved and divergent patterns of gene expression *P. falciparum* single-cell data was integrated with the *P. berghei* transmission stage data using one-to-one orthologs. **a**–**c** UMAP of integrated data colored by species (**a**), collection stage (**b**), and cluster assignment (**c**). Collection stages that were not included in the *P. berghei* data set are marked in legend with (*Pf*) in (**b**). Cluster C1 was not included in further differential expression analysis because it was composed primarily of *P. falciparum* oocyst and hemolymph sporozoites. We observed that cells clustered by cell types that were shared across the two data sets. *P. falciparum* gametocytes appeared more tightly clustered compared to *P. berghei*, within the gametocyte clusters, likely owing to differences in sample sizes for these stages (Pb female: 127, Pb male: 74, Pf female: 52, Pf male: 33). **d** Expression (log_2_ counts) of the top-conserved marker gene, based on the lowest maximum *p* value (Wilcoxon rank-sum test) across the two species, for clusters 0 (SLARP), 2 (Cap380), 3 (conserved *Plasmodium* protein PF3D7_0510700), and 4 (calmodulin-like protein) on the UMAP. **e** Heatmap of the top five conserved marker genes based on Bonferroni-adjusted *p* value (Wilcoxon rank-sum test) across the two species, for each cluster. The expression values represent the mean log counts for each cluster. **f** A heatmap of the top one-to-one orthologs that are more highly expressed in *P. falciparum (*Pf*)* compared with *P. berghei (*Pb*)* (based on adjusted *p* value and expression in at least 70% of *P. falciparum* cells). The expression values represent the mean log counts for each species in each cluster. Heatmaps are annotated with the *P. falciparum* gene identifier for the ortholog and manual annotation. Genes annotated as conserved are conserved proteins with unknown function in *Plasmodium*.

To further explore the conservation of genes involved in *Plasmodium* transmission, we compared *P. falciparum* transmission stages with the equivalent *P. berghei* stages from Howick et al.^25^ using scmap. The two data sets showed a good overall correlation, with *P. falciparum* cells mapping to cells of the same stage in the *P. berghei* reference data set (Supplementary Fig. 13a, b)^8^. As expected, cells that were not represented in the *P. berghei* transmission analysis (early sporozoite developmental stages), showed lower similarity across species than those that were common to the two data sets (Supplementary Fig. 13c). This initial comparison enabled us to assert that differences between experimental workflows, which in our case were optimized to purify non-genetically modified parasites and thus differed slightly from those used by Howick et al.^12,13^ were not a likely source of variability between the data sets. We next used an unsupervised integration and graph-based clustering approach in Seurat to elucidate gene expression patterns across the transmission stages of the two species. Using this approach, cells from the two data sets could be matched to four clusters, with cells mixing by stage in the life cycle (Fig. 2a–c). A fifth cluster (C1) was populated by *P. falciparum* midgut and hemolymph sporozoites that were matched by very few cells in the *P. berghei* data set (Fig. 2a–c). The global structure of the integrated data set remained largely unchanged compared with the original *P. falciparum* subset, underlining the robustness of the cell identity correlations. For each cluster, we identified marker genes that are conserved between species (Fig. 2d, e). Among genes that represent the conserved transcriptome (Fig. 2d, e; Supplementary Data 1), 30–40% have no known function, but as exemplified for the ookinete subset (Supplementary Fig. 6f), we can begin to make predictions about gene function by surveying correlated gene expression patterns along highly resolved developmental trajectories. The same strategy could help to fast-track the discovery of candidates for therapeutic development, for example, by uncovering genes with expression profiles that are highly correlated with those of validated vaccine targets (e.g., Pfs25, CSP)^26^.

In addition to shared patterns in genome activity with broadly conserved *Plasmodium* genes, our integrated analysis also revealed non-conserved patterns of gene expression. In all stages, we identified genes whose transcripts could be detected in over 70% of *P. falciparum* cells but showed no or only residual expression in *P. berghei* cells of the same stage (Fig. 2f; Supplementary Data 1). Parallel analysis of previously published asexual blood-stage transcriptomes from both species^6,8^, as well as different strains of *P. falciparum*^27^, showed that genes that were differentially expressed between species were not *P. falciparum* strain-specific in expression (Supplementary Fig. 14). This suggests that species-specific expression patterns observed in transmission stages are not driven by intra-species genotypic variation in gene expression. Together, with the genes that do not have orthologs in *P. berghei* (Supplementary Fig. 11a, b; Supplementary Data 1), these differentially expressed genes may represent species-specific adaptations that allow *P. falciparum* to interact optimally with the diverse host environments it meets as it transmits from vector-to-host and vice-versa. For example, *P. falciparum* ookinetes (C2_Pf in Fig. 2f) displayed a striking pattern of species-specific expression with 26 genes (several of which have no known function) expressed in >70% of *Pf* cells and in <10% of *Pb* cells (Supplementary Data 1). Conversely, in sporozoites, a single one-to-one ortholog—encoding a putative mRNA methyltransferase (PfMT-A70.2)—showed *P. falciparum-*specific expression (Fig. 2f). PfMT-A70.2 was recently identified as part of a methyltransferase complex that modulates gene expression during the IDC through N^6^-methyladenosine methylation of mRNA transcripts^28^. Our observation that PfMT-A70.2 expression is highly correlated with mosquito stages in *P. falciparum* but not in *P. berghei* (Fig. 2f), hints at unexplored species-specific mechanisms to fine-tune gene expression during the parasite journey in the mosquito.

### Trajectory analysis of sporozoite development identifies a putative egress network

**Fig. 3:**
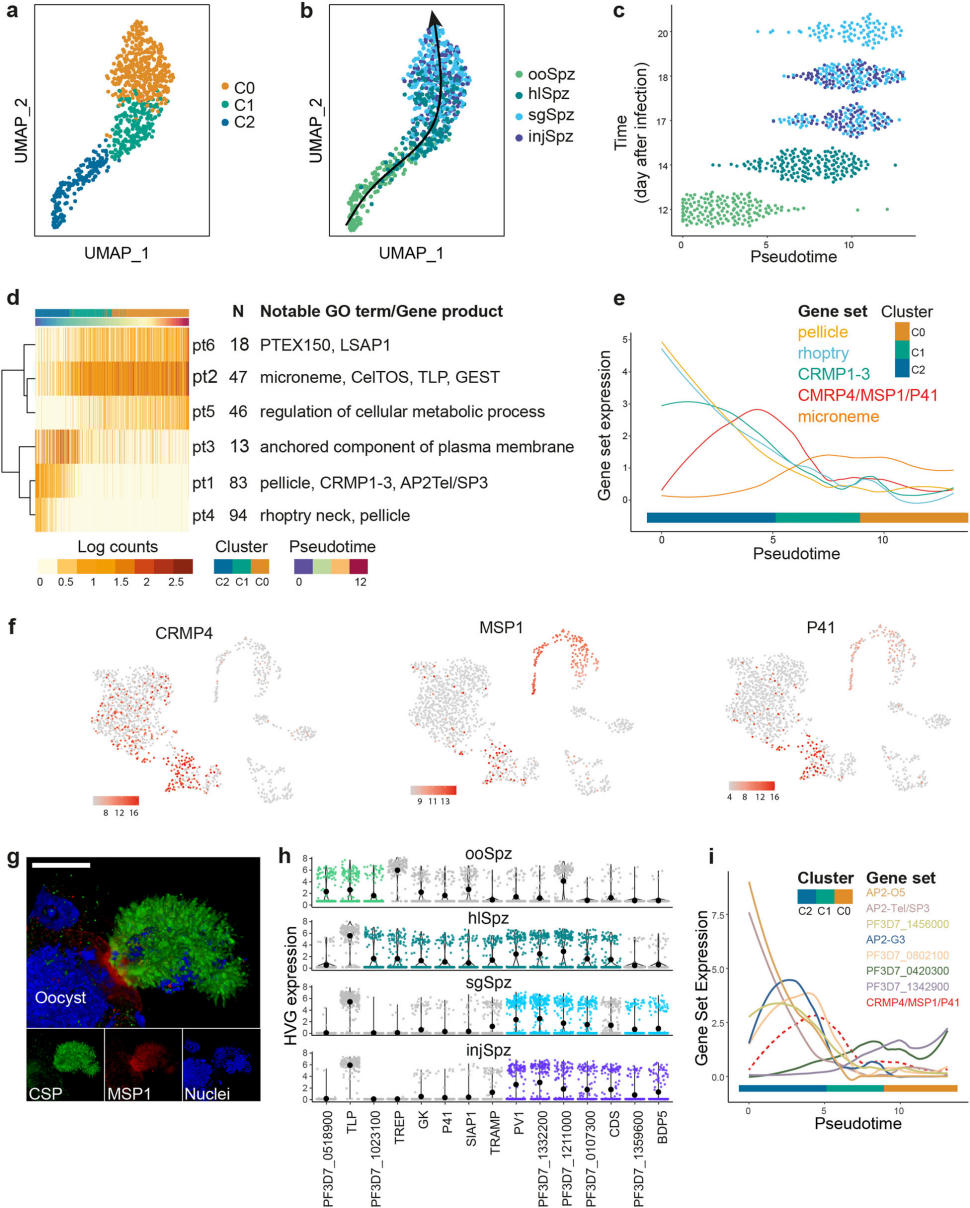
The sporozoite journey in the mosquito. **a**, **b** UMAP representation of single-cell sporozoite transcriptomes at different stages of the sporozoite developmental journey colored by cluster assignment (**a**) and sorted stage (**b**). The arrow in B represents the direction of development. **c** Sporozoites aligned along pseudotime. Overall, the pseudotime trajectory correlates with the direction of development indicated by the time of sample collection in the *y* axis. **d** Heatmap showing the mean expression of differentially expressed (DE) genes over pseudotime, arranged into six pseudotime clusters (pt1–pt6). Each row in the heatmap represents the average expression of all genes in one cluster (the number of genes in each cluster, *N*, is indicated). Genes in each cluster show similar expression dynamics along the developmental trajectory. **e** Gene modules representative of enriched GO terms (see Supplementary Data 4 for Bonferroni-adjusted *p* values) in pseudotime clusters are shown. Transient expression of CRMP4, p41, and MSP1 (GO term anchored component of plasma membrane in **d**) correlates with the release of sporozoites into the hemocoel. **f** UMAP representations of the complete life cycle (as shown in 1b) with the expression (log counts) of CRMP4, p41, and MSP1 highlighted. **g** 3D rendering of a 12-day oocyst immunostained with anti-CSP (green), anti-MSP1 (red), and Hoechst (blue). The image captures the egress of sporozoites (CSP positive) from the oocyst. Released sporozoites express MSP1 on their surface. Note that the lack of signal in sporozoites that remain inside the oocyst is likely a result of poor antibody penetration across the oocyst wall. Scale bar, 10 µm. The image is representative of 6 egress events (out of 9), each releasing hundreds of sporozoites, captured from 19 infected midguts in two independent infections. **h** Expression of highly variable genes (HVG) across sporozoite developmental stages. HVGs were independently determined for each stage using M3Drop with a false discovery rate (FDR) < 0.05. Only HVGs that are also associated with the pseudotime trajectory are shown. HVGs are colored by their stage association, as per (**b**). **i** Expression of DE ApiAP2 TFs over pseudotime. AP2-Tel/SP3 and AP2-O5 expression levels steadily decline from an earlier peak in ooSpz until egress (marked by the C2–C1 transition and expression of CRMP4/MSP1/P41). Three ApiAP2 TFs are transiently expressed before egress, whereas another two are upregulated after egress.

Understanding how sporozoite infectivity towards the human host develops is likely to inform the identification of untested antimalarial and vaccine candidates. We, therefore, sampled these stages more densely to capture a finely resolved developmental trajectory that recapitulated the sporozoite journey through the mosquito, with its different anatomical, cellular, and physicochemical environments^29^. We initially re-clustered sporozoite cells and confirmed through differential abundance testing that cluster composition was not affected by experimental batch effects (Supplementary Fig. 15). Next, sporozoites were oriented along a pseudotime trajectory where the relative ordering of each individual cell was defined by its transcriptional state (Fig. 3a, b; Supplementary Data 2). This enabled us to explore fine transcriptional changes associated with a continuum of development instead of discrete time points, which were observed to be heterogeneous (Fig. 3c). The pseudotime trajectory aligned with the expected direction of development, with cells broadly segregating by their anatomic site and developmental time point (Fig. 3b, c). Differential expression (DE) analysis uncovered 301 genes with expression significantly associated with pseudotime (*q* value < 0.0001; Supplementary Data 1). Approximately 20% of these DE transcripts overlapped with genes previously identified as upregulated in oocyst sporozoites (UOS) or upregulated in infectious sporozoites (UIS) (Supplementary Fig. 16a, b; Supplementary Data 1)^30^, but many others have never been linked with sporozoite biology.

To reveal patterns of gene activity along the pseudotime trajectory, we grouped genes with similar expression dynamics into co-expression modules (denoted pt1–6, Fig. 3d; Supplementary Data 1). This analysis revealed two largely mutually exclusive transcriptional programs, with the midgut-to-hemocoel transition (C2– > C1) marking an abrupt shift in gene expression. Gene modules (pt1,4) featuring known salivary gland invasion genes (Cysteine Repeat Modular Protein, CRMP1-2)^31^, components of the rhoptry neck and inner membrane complex were the first to downregulate expression. This was followed by a transient peak (pt3) showing high temporal correlation with sporozoite release into the hemocoel and preceding the transcriptional changes that characterized later development (Fig. 3d, e). Of note, one of the 13 genes that constitute the pt3 module, CRMP4, has been linked to sporozoite egress in *P. berghei*^32^. Two other genes within this module—MSP1 and the 6-Cys family member p41^33,34^—encode merozoite surface proteins that have never been associated with mosquito stages. Although the function of p41 has not been elucidated, MSP1 facilitates merozoite egress by destabilizing the erythrocyte submembrane cytoskeleton^35^. Its co-regulation with CRMP4 suggests an unexpected mechanistic link between sporozoite and merozoite egress (Fig. 3f). The existence of a functional link to egress is further supported by evidence that MSP1 is expressed on the surface of sporozoites at the time of their release from the oocyst (Fig. 3g; Supplementary Movie 1), which not only validates our scRNA-seq findings but also places MSP1 within the right spatial and temporal window for such a role. Given their highly correlated expression, we hypothesize that genes in this module form the core of a sporozoite egress functional network and anticipate that future studies into the role of MSP1 may uncover parallels between events that culminate in the rupture of the oocyst wall and that of the erythrocyte membrane.

### HVGs highlight heterogeneity of sporozoite populations

After their release into the hemocoel, sporozoites experienced a dramatic shift in gene expression. Several transcripts previously identified as UIS^30^ begin to be expressed during this stage or, in some cases (e.g., CelTOS, TLP), even before egress (Fig. 3d; Supplementary Fig. 16a; Supplementary Data 1). Approximately half of the transcripts expressed or upregulated after egress are absent from recently published sporozoite proteomes (Supplementary Fig. 16d; Supplementary Data 1)^30^, which would suggest that at least some might be under translational repression, although further work will be necessary to determine case-by-case, if lack of detection in proteomics studies truly represents the absence of protein expression. A small proportion (6%) of developmentally regulated transcripts show highly variable expression between individual parasites, with salivary gland and injected sporozoites displaying significant cell-to-cell heterogeneity in the expression of genes implicated in protein export to the host cell (PV1^36^) and epigenetic mechanisms (BDP5^37^), amongst others (Fig. 3h; Supplementary Data 1). A pairwise correlation analysis did not find evidence of co-regulated expression of HVGs, suggesting that HVGs are not co-transcribed in a subset of sporozoites, and, instead, individual sporozoites activate the expression of different HVGs (Fig. 3h; Supplementary Fig. 17; Supplementary Data 1). Given the strong association of these HVGs with sporozoite development, it is tempting to speculate that the observed differences in expression between individual sporozoites could result in different readiness levels with respect to navigating the journey to the liver and/or establishing a new replication niche once they have reached their destination.

### Trajectory analysis implicates multiple ApiAP2 transcription factors in sporozoite development

Consistent with the key role of ApiAP2 TFs in driving all major developmental switches during the parasite life cycle^38^, seven ApiAP2 transcripts were found to be dynamically regulated along the sporozoite developmental trajectory (Supplementary Fig. 18). Of the five ApiAP2 TFs expressed during late midgut sporogony (cluster C2), only one—PfAP2-Tel/SP3 (PF3D7_0622900)—had been previously implicated in sporozoite development. Gene knockout studies in *P. berghei* showed that AP2-Tel/SP3 has a role in sporozoite release^39^ and indeed, we observed that PfAP2-Tel/SP3 expression preceded the transcription of egress genes (Fig. 3i). Future studies should examine whether PfAP2Tel/SP3 has a role in regulating the expression of egress-related genes and to what extent recent findings mapping PfAP2Tel/SP3 to the telomeric ends of *P. falciparum* chromosomes^21^ might be linked to this putative activity. In contrast to PfAP2-Tel/SP3, AP2-G3. and AP2-O5, which have known roles in gametocytogenesis and in regulating ookinete motility, respectively, have not been previously implicated in sporozoite development^40^. Together with AP2-G3, two CA-repeat-binding ApiAP2 transcription factors^41^, PF3D7_0802100, and PF3D7_1456000, show a peak in expression prior to egress (Fig. 3i; Supplementary Fig. 18), raising the possibility that all three might regulate the expression of genes necessary for sporozoite release or, alternatively, drive the transcriptional response post egress. Expression of the putative transcription factors PF3D7_0420300 and PF3D7_1342900, in contrast, did not occur until after egress (Fig. 3i; Supplementary Fig. 18). Attempts to knock out the former in *P. falciparum* have previously proved unsuccessful, suggesting PF3D7_0420300 has an essential role in asexual development^16^. How ApiAP2 TFs active in multiple stages of the life cycle achieve stage-specific outcomes is not well understood but may involve combinatorial binding between members of the ApiAP2 family^38^. It is noteworthy that ApiAP2 TF expression patterns during sporogony are consistent with the AP2 TF cascade model, whereby ApiAP2 TFs are themselves under the control of other ApiAP2 TFs. Thus, our analysis expands the constellation of ApiAP2 TFs with putative roles in sporozoite development and suggests that a far more complex regulatory network than previously thought—involving specific combinations of ApiAP2 TFs possibly acting cooperatively—might be required to orchestrate the dynamic transcriptional patterns associated with the sporozoite journey in the mosquito.

### A shift in the host environment corresponds with gradual changes in gene expression

**Fig. 4:**
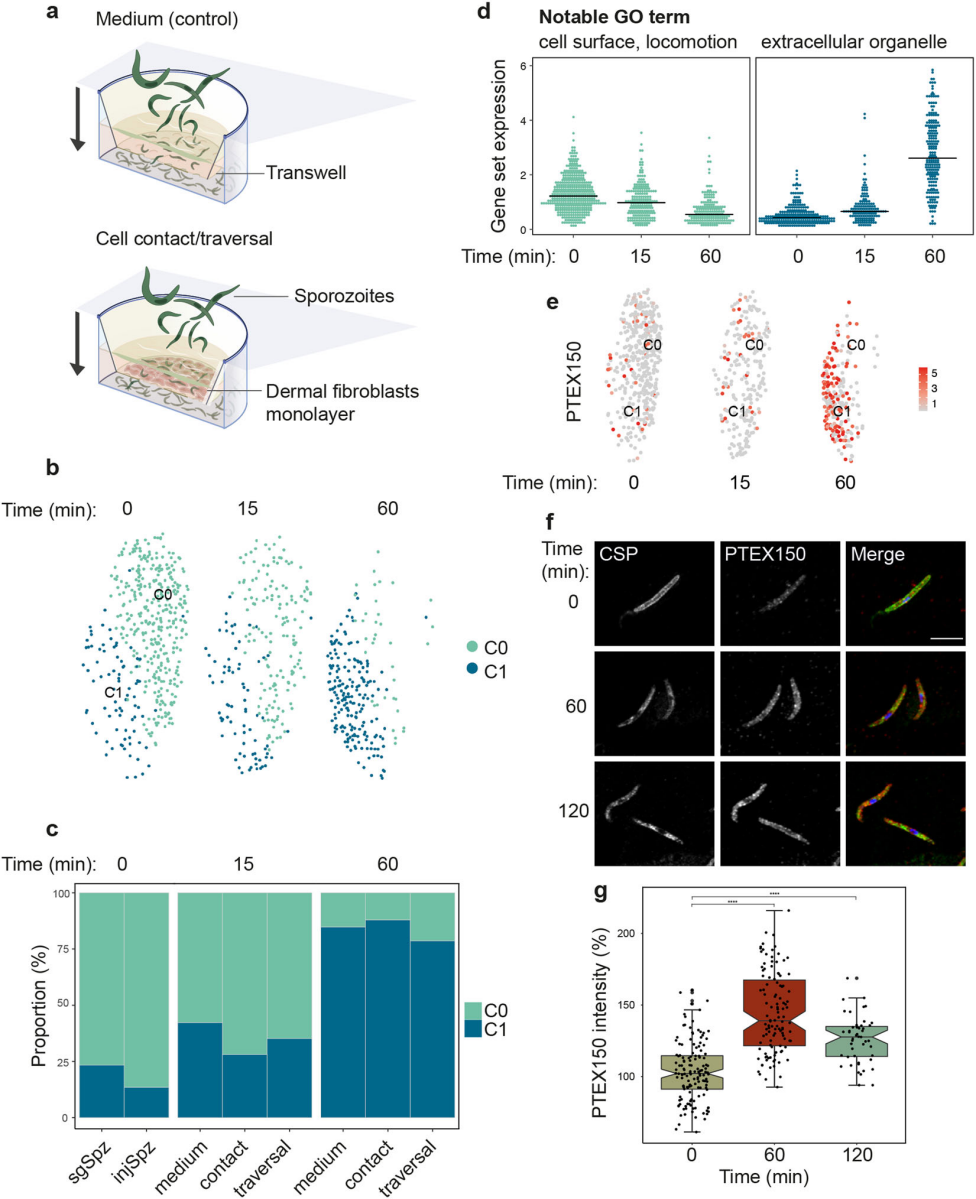
Sporozoite activation in the mammalian microenvironment. **a** Schematics of the transwell sporozoite activation assay. The skin environment was reproduced by transferring sporozoites to transwell inserts filled with fibroblast growth medium and coated with human primary fibroblasts or kept cell-free at 37°C. Sporozoites were allowed to interact with this setup for 15 or 60 min, after which they were collected from both the top and bottom compartments of the transwell. To reach the bottom compartment in the presence of the fibroblast monolayer sporozoites had to deploy their cell traversal activity, as the transwell pores were blocked by the tight cell monolayer. “Medium” refers to sporozoites exposed to medium alone, “contact” refers to sporozoites that were collected above the cell monolayer, and “traversal” refers to sporozoites that passed through the cell monolayer and were collected from the bottom compartment. Salivary gland sporozoites kept in insect medium and injected sporozoites were considered as time 0. **b** UMAP representations of sporozoite transcriptomes colored by cluster assignment before and after activation. **c** Quantification of sporozoite activation based on the cluster assignments in (**b**). **d** Average expression of down- and upregulated genes during sporozoite activation. Each panel comprises all DE genes that present a fold change of at least 1.5 between C0 and C1 and are expressed in >25% of the cells (Supplementary Data 1). The top GO terms in each module are indicated (GO:0009986, GO:0040011, GO:0043230) and the corresponding Bonferroni-adjusted *p* values are summarized in Supplementary Data 4. **e** UMAP plots of single-cell sporozoite transcriptomes with the expression of PTEX150 before (0 min) and after activation (15 and 60 min) shown. Expression levels (log_2_ counts) are color-coded as per the scale. The cell cluster assignments from (**b**) are indicated. **f** Expression of PTEX150 protein in *P. falciparum* sporozoites before and after activation. Sporozoites were fixed at the indicated time points and immunostained with anti-CSP (green), anti-PTEX150 (red), and Hoechst (blue). Scale bar, 5 µm. **g** Quantification of PTEX150 protein levels. The boxplot shows normalized mean fluorescence intensities, with the notch representing the 95% confidence interval of the median and whiskers denoting the data range within 1.5× of the interquartile range. A total of 140, 114, and 47 individual sporozoites were analyzed at 0, 60, and 120 min, respectively. For 0 and 60 min, *n* = 3 independent experiments; for 120 min, *n* = 1 with two replicates. Statistical significance was assessed using one-way ANOVA with Tukey multiple corrections. ****0 min vs 60 min, *p* = 3.27 × 10^−28^; 0 min vs 120 min, *p* = 6.45 × 10^−11^.

As sporozoites migrate away from the site of injection, they can spend up to 2 hours probing their environment, actively migrating in and out of dermal fibroblasts and phagocytic cells in search of a blood vessel through which they can gain access to the bloodstream^42–44^. Being devoid of a protective vacuole, sporozoites migrating in the dermis are particularly exposed to the onslaught of host immunity^45^. Indeed, the most advanced efforts at inducing immunity against pre-erythrocytic parasite stages have targeted the sporozoite in the skin^26^. Despite this, the skin stage of the sporozoite journey remains poorly understood and the effects of the dermal microenvironment and cell traversal on the transcriptional activity of the sporozoite have yet to be explored. To capture this elusive transcriptome, we allowed salivary gland sporozoites to migrate through transwell µ-pores coated with a monolayer of primary skin fibroblasts, before collecting sporozoites that had either traversed or simply contacted the cell monolayer (Fig. 4a). Although our clustering and differential gene expression analysis revealed that sporozoites adapted their transcriptional activity to cues from the host-like microenvironment, medium-only controls were sufficient to induce a gradual shift in gene expression and neither contact with dermal fibroblasts nor cell traversal was found to have an additional impact (Fig. 4b, c). The observed changes (Supplementary Data 1) were consistent, although not as wide-ranging, with the documented shifts in gene expression experienced by sporozoites exposed to host-like conditions for prolonged periods of time^46^ (Supplementary Fig. 19; Supplementary Data 2). This suggests that host-induced changes in transcription may occur gradually in a sequential fashion. After 1 h, salivary gland sporozoites had downregulated the expression of several genes known to be involved in cell traversal (GEST, SPECT1, PLP1, TLP)^44,46–48^. Concomitantly, a cohort of genes associated with protein folding^49^ and export^36,50^, including HSP70/90, PTEX150, and PV1, was upregulated (Fig. 4d, e; Supplementary Data 1). PTEX150, which has not been previously implicated in sporozoite biology, is a core component of the *Plasmodium* translocon of exported proteins (PTEX) complex^50^, responsible for protein trafficking into the host cell following merozoite invasion. Upregulation of PTEX150, which we confirmed by immunofluorescence analysis (Fig. 4f, g), occurred within a timeframe when sporozoites are still en route to the liver, suggesting that protein export to the host cell might be one of the earliest events following the productive invasion of a hepatocyte. This would mirror merozoite invasion, where the PTEX complex is inserted into the newly formed parasitophorous vacuole membrane within minutes^51^, enabling the export of effector proteins into the erythrocyte to build a suitable niche for replication and immune evasion^52^. It is worth noting that this activated phenotype was also observed in a small proportion of salivary gland sporozoites before their ejection from the mosquito (Fig. 4b, c, e). Whether this is the result of spurious signaling events that disrupt sporozoite latency, or instead represents a developmentally regulated process that renders sporozoites transmission-ready awaits to be established.

## Discussion

Here, we present a comprehensive single-cell analysis of the transcriptional journey of the human malaria parasite *P. falciparum* through its transmission cycle. The transcriptomes presented here provide an in-depth characterization of gene expression across distinct phenotypic stages of development and add a chapter to the Malaria Cell Atlas, which is an interactive data resource supporting the exploration of individual *Plasmodium* parasite transcriptomes now across the life cycle from two malaria parasite species. This data set captures key aspects of parasite development within the mosquito and beyond, providing insights into how those developmental processes may be regulated, as well as elucidating potential targets for disrupting the transmission cycle to control malaria. Exploration of correlated gene expression trajectories within this data set can be the starting point to discover the function of genes that lack functional annotation or reveal unexpected potential functions for other more well-studied genes. For example, our finding of a possible role for MSP1, a vaccine candidate implicated in merozoite egress, in the release of sporozoites from the oocyst to the mosquito hemocoel, suggests that a conserved egress machinery may be deployed at multiple points in the life cycle. Another example of note is that of PTEX150, whose pattern of expression following sporozoite activation suggests an early role in liver stage biology. Recent observations that EXP2, the pore-forming component of the PTEX complex, is necessary for sporozoite invasion of hepatocytes^53^ together with the fact that PTEX150 acts as an adaptor between EXP2 and a third translocon component, HSP101^54^, raise the possibility that PTEX150 might play a role in or shortly after hepatocyte invasion as part of the PTEX translocon complex. Strategies that target this complex might thus be effective at disrupting asexual replication as well as blocking the onset of liver stage infection. More generally, the global view of gene usage across the life cycle made possible by the Malaria Cell Atlas sheds light on core functional strategies that will help inform the development of previously unappreciated multistage antiplasmodial interventions. Such multistage functional classes represent attractive targets for drug development or other interventions aimed at both the host and the mosquito vector.

Transmission of parasites between humans and mosquitoes represents the major population bottlenecks in the malaria life cycle^1^. After an infectious blood meal, developmental transitions within the mosquito midgut are accompanied by a steep reduction in parasite numbers (down to the tens of units). At the other end of the transmission cycle, not only is the number of sporozoites injected into the skin during a bite generally low, but an even smaller number find their way out of the site of injection and reach the liver to initiate a new phase of population expansion within a hepatocyte^45^. The factors that determine whether an individual parasite will survive this journey are unclear, but one intriguing possibility is that the ability to interact with and respond to the host environment in a productive manner could vary from parasite to parasite. Of key importance, we identify developmentally regulated transcripts that show highly variable expression across cells of the same stage and could theoretically account for differential phenotypes within a population. Amongst the genes with variable expression in salivary gland sporozoites is PV1, a translocon accessory protein^36^ that is also a hallmark of activated sporozoites. It is thus tempting to speculate that sporozoites that express PV1 prior to the transmission might have a competitive advantage compared with the wider majority that does not activate PV1 expression until transmission to the host has occurred. Transcriptional heterogeneity within sporozoite populations has also been observed in a study of *P. berghei* mosquito stages published while this work was under consideration^55^. Although it is not clear whether population heterogeneity is driven by the same highly variable transcripts in the two species, it appears that transcriptional heterogeneity is a conserved feature of sporozoite development. Phenotypic validation may reveal whether there is a transcriptional signature that correlates with infectiousness and predicts the likelihood of successful transmission. Further interrogation of this data set will serve as a major accelerator for the discovery of future targets to block human malaria transmission and ultimately prevent the establishment of malaria disease.

## Methods

### Parasite maintenance and isolation

#### *P. falciparum* maintenance and gametocyte induction

*P. falciparum* gametocytes were induced and cultured as before^56^. In brief, asexual blood-stage cultures were maintained in asexual culture medium (Roswell Park Memorial Institute (RPMI) 1640 with 25 mM 4-(2-hydroxyethyl)-1-piperazineethanesulfonic acid (HEPES) (Life Technologies), 50 µg L^−1^ hypoxanthine (Sigma), 0.3 g L^−1^ l-glutamine (Sigma), and 10% A + human serum (Interstate Blood-Bank)) with a hematocrit (HC) of 5%. Gametocyte cultures were induced at 2.5% parasitemia and maintained for 15–17 days in gametocyte culture medium (RPMI 1640 with 25 mM HEPES (Life Technologies), 50 µg L^−1^ hypoxanthine (Sigma), 2 g L^−1^ sodium bicarbonate (Sigma), 0.3 g L^−1^ l-glutamine, 5% A + human serum (Interstate Blood-Bank) and 0.5% AlbuMAX II (Life Technologies), 5% HC) with daily media changes.

#### Standard membrane feeding assay

*Anopheles stephensi* mosquitoes were reared under standard conditions (26–28 °C, 65–80% relative humidity, 12:12 h light/darkness photoperiod). Adults were maintained on 10% fructose, except for the 12 h before an infectious blood meal, when mosquitoes were starved. Mosquito infections were carried out using a standard membrane feeding assay (SMFA), as previously described^57^. In brief, 15–17-day-old gametocyte cultures with at least 2% stage V gametocytaemia were diluted in a 50% mixture of fresh RBCs and A + human serum and then used to feed starved female mosquitoes. Mosquitoes were allowed to feed for 12 min, after which they were kept without fructose for 24 h, so that only blood-fed mosquitoes would survive. From then on, mosquitoes were given fresh fructose daily until the day of dissection.

#### Retrieval of gametocytes

At day 16 post induction, gametocytes were loaded on a 70% Percoll gradient and spun at 500 × *g* for 10 min without brake at 38 °C. Gametocytes were washed once in gametocyte medium at 38 °C and the pellet resuspended in 1 ml of suspended animation (SA) buffer^58^ supplemented with 2 μg/ml Hoechst 33342 and 4 μg/ml Pyronin Y. In the presence of Hoechst 33342, interactions between Pyronin Y and DNA are disrupted and Pyronin Y mainly stains RNA^59^. Gametocytes were stained at 4 °C for 20 min. After 20 min, cells were washed twice in SA and resuspended in fresh SA, passed through a strainer with single gametocytes sorted into a 96-well plate using a BD FACsAriaIII at 4 °C. PyroninY was detected using a 561 nm laser with a 586/16 nm filter, and Hoechst 33342 was detected using the 488 nm laser with the 450/50 nm filter (Supplementary Fig. 2). Gametocytes were collected from parasites deriving from either an NF54 genetic background (African) or a recent, culture-adapted field isolate, APL5G (Cambodian)^60^.

#### Retrieval of ookinetes

In all, 24 h after SMFA, mosquitoes were dissected, and the blood bolus removed from the midguts. The collected blood were placed into ice-cold PBS supplemented with 2× protease inhibitors (Halt™ Protease Inhibitor Cocktail, Thermo Fisher) and 50 mM ethylenediaminetetraacetic acid. For the first pilot flow cytometry experiment (Supplementary Fig. 1A), uninfected RBCs and activated gametocytes cultures were placed in ookinete medium (RPMI-HEPES supplemented with 100 µM xanthurenic acid, 200 µM hypoxanthine, and 10% bovine serum albumin, pH7.4) for antibody- and DNA staining and used as controls. Each sample was split into four aliquots, one aliquot remained unstained and the others were incubated with either 1:500 anti-Pfs25-cy3 antibody^56^, 1:1000 DRAQ5 DNA stain (BioLegend), or both, respectively, at 4 °C in the dark for 30 min. Samples were then washed twice in ice-cold PBS and resuspended in ice-cold PBS, before being analyzed on a BD Fortessa using the 561 nm laser and filter 586/15 for cy3 and the 640 nm laser and 730/45 filter for DRAQ5, respectively. For the sorting, ookinetes collected 24 and 48 h after a feed were prepared as above and sorted into a 384-well plate. One-fifth of the plate was sorted from the P1 population and the rest from the P2 population (Supplementary Fig. 1B, C). Oocysts per midgut were counted on day 10 post-feed from 41 mosquitoes and revealed a prevalence of infection of 63%.

#### Retrieval of sporozoites

Sporozoites were collected on different days from three anatomic sites in the mosquito to capture different stages of development. sgSpz were collected on three different days, representing three independent infections. injSpz originated from two independent infections, whereas ooSpz and hlSpz derived from a single experiment. Before dissections, mosquitoes were cold anesthetized (4 °C) and killed inside a secure glove box by beheading. Oocyst and salivary gland sporozoites were obtained by dissecting mosquito midguts and salivary glands, 12 and 17–20 days post blood meal, respectively. Hemolymph sporozoites were released from mosquito carcasses on day 14 after removal of both salivary glands and midguts. All samples were initially homogenized with a disposable pestle to release sporozoites from the mosquito tissues. After a filtration step with a 20 µm filter (Pluriselect), sporozoites were loaded onto a 3 ml Accudenz cushion and centrifuged at 2500 × *g* for 20 min^61^. All the above procedures were carried out at 4 °C in Schneider’s insect medium. After centrifugation, sporozoites were collected from a thin layer on top of the Accudenz cushion (mosquito debris separated to the bottom), washed once with cold Schneider’s buffer (12,000 × *g*, 3 min, 4 °C), and resuspended in the same buffer supplemented with 1% fetal bovine serum (FBS). Before FACS sorting, sporozoites were immunostained for 30 min with anti-CSP (2A10 clone, MR4) diluted at 1:200, followed by a 20-minute incubation with an Alexa Fluor 488-conjugated anti-mouse antibody (1:300, Jackson ImmunoResearch) and DRAQ5 (1:1000, BioLegend). All incubations and washes were carried out at 4 °C. Single cells were sorted directly into NEB lysis buffer on a BD FACSAria III equipped with a 100-µm nozzle.

Sporozoites released through a mosquito bite were collected by allowing 300–500 mosquitoes to feed on a warm fructose solution (80 g/L) supplemented with 10% serum for 5 min. After confirming that sporozoites had been released and that no mosquito debris were present in the sample, sporozoites were stained for 20 min with Draq5 at 4 °C and sorted directly into NEB lysis buffer.

#### Cell traversal assay

Sporozoites released from salivary glands on day 20 after a blood meal were purified as before and resuspended in fibroblast growth medium (PromoCell) supplemented with heat-inactivated bovine serum (Sigma) to a final concentration of 10%. In all, 50,000 sporozoites were added per well of a 24-well plate fitted with Transwell inserts (5 µm pore size, Corning) coated with 100,000 human primary dermal fibroblasts (PromoCell) to form a tight monolayer. Sporozoites were allowed to migrate for 15 or 60 min and were then recovered separately from the top and bottom Transwell compartments. On average, 35% of the input was recovered from the bottom Transwell compartment. Some control wells did not contain fibroblasts, so that the effect of the medium on the sporozoites could be evaluated. Before FACS sorting, sporozoites were immunostained as described above, with all steps carried out in fibroblast growth medium with 10% FBS at 4 °C. Single cells were sorted as before.

#### Immunofluorescence analysis

Sporozoites were isolated as described above and resuspended in Dulbecco’s modified Eagle’s medium with or without supplementation with heat-inactivated bovine serum (Sigma), at either RT or 37°C. At the indicated time points, 50,000 sporozoites were spun down (3000 rpm, 5 min) on a glass coverslip coated with poly-l-lysine (Sigma) and allowed to settle for 10 min before fixation with 4% paraformaldehyde (PFA, Pierce) for 20 min. Sporozoites were then permeabilized for 5 min with ice-cold methanol (Sigma) and blocked for 1 h with a solution of 5% normal donkey serum (Jackson ImmunoResearch) and 0.01% Triton X-100 (Sigma) in PBS. Primary antibodies were diluted in blocking solution and incubated overnight at 4 °C. Anti-PTEX150 was a kind gift from Paul Gilson (1:500, Burnet Institute, Australia) and anti-CSP (1:200, clone SAI131-5D5) was obtained from MR4. After three washes with blocking solution, samples were incubated with AlexaFluor-conjugated secondary antibodies (1:500, Jackson ImmunoResearch) and Hoechst 33342 (Invitrogen) for 1 h at room temperature (RT) and washed again. The coverslips were then mounted on microscope slides using Fluoromount-G (Thermo Fisher) and imaged with a Leica SP8 inverted confocal microscope equipped with a ×63 objective. For quantification of PTEX150 expression in sporozoites, images were processed and analyzed using custom macros in Fiji version 2.1.0/1.53c.

For imaging oocysts, the midguts of infected mosquitoes were dissected on day 12 post infection and fixed with 4% PFA for 1 h at RT. Samples were permeabilized with acetone for 5 min, washed, and blocked overnight at 4 °C with 5% normal donkey serum, 0.1% Triton X-100 in PBS. Primary antibodies (1:500 rabbit anti-MSP1-19, a gift from Paul Gilson; 1:200 mouse anti-CSP, MR4) were diluted in blocking solution and incubated overnight at 4 °C. After three 15-minute washes in blocking solution, samples were incubated with AlexaFluor-conjugated secondary antibodies (1:500, Jackson ImmunoResearch) and Hoechst 33342 (Invitrogen) for 1 h at RT. Following two more washes in blocking solution and a final wash in PBS, midguts were mounted on microscope slides using Fluoromount-G. Z-stack images were acquired on a Leica SP8 inverted microscope equipped with a ×63 objective and processed using Volocity software.

### scRNA-seq

Cells were sorted in 96- or 384-well plates on FACSAria III into NEB lysis buffer (0.1× cell lysis buffer E6428B-SG, 0.05× RNAse-inhibitor Murine E6429B-SG, and 0.85× nuclease-free water E6433B-SG, from New England Biolab). The ookinetes were sorted into a 384-well plate, and all other samples were sorted into 96-well plates. The 384-well plate contained 2 µL lysis buffer, whereas the 96-well plates contained 4 µL. Reverse transcription and PCR were performed in the Sanger Institute single-cell sequencing pipeline using the NEBNext Single Cell/Low Input RNA Library Prep Kit for Illumina (E6420L) with 26 PCR cycles. cDNA was purified using an AMPure XP bead clean-up on either an Agilent Bravo or Hamilton Star. Reaction volumes were doubled in line with the starting volume of the lysis buffer in 96-well plates for cDNA generation, amplification, and purification. The samples were quantified using the Mosquito and Omega plate reader to calculate the volume of cDNA needed for library preparation. Libraries were made using the scRNA cDNA-XP plates, bravo platforms, and NEB Ultra II FS reagents (E7805L). Libraries had an additional eight cycles of PCR. Libraries were then pooled to 384 samples prior to AMPure XP bead Clean-up on a Hamilton Star. The samples were run on an Agilent Bioanalyser and normalized to 32.8 nM prior to sequencing. Samples were sequenced on a HiSeq 2500 with 75 bp paired-end reads.

### scRNA-seq mapping and analysis

#### Single-cell transcriptome mapping and read counting

Data acquisition and mapping followed the pipeline described in ref. ^6^. In brief, reads were trimmed using trimgalore (cutadapt v 1.18)^62^. Trimmed reads were mapped to the *P. falciparum* v3 genome (https://www.sanger.ac.uk/resources/downloads/protozoa/ October 2016) with HISAT2 (v 2.1.0)^63^ using *hisat2 --max-intronlen 5000 p 12*. Mapped reads were then summed against transcripts with featureCounts in the Subread package (v 2.0.0)^64^ using *featureCounts -p -t CDS -g transcript_id*.

#### Quality control and normalization

Given the high variability of genes detected across the life cycle^8^, low-quality cells were identified based on the distribution of the number of reads and genes detected per cell within each parasite stage (gametocytes, ookinetes, sporozoites). For gametocytes, cells with fewer than 500 genes and 10,000 reads per cell were removed. For ookinetes, cells with fewer than 400 genes and 5000 reads per cell were removed. For sporozoites, cells with fewer than 40 genes per cell and 5000 reads per cell were removed (Supplementary Fig. 4, Supplementary Table 1). Transcriptomes were normalized using a deconvolution size factor with scran (v 1.16.0)^65^ using quickCluster() and calculateSumFactors() to account for the large differences in detection between parasite stages. Visual inspection of the cell-wise relative log expression plots (Supplementary Fig. 4) shows normalization by scran smoothed profiles across stages.

#### Cell clustering and projection

To delineate parasite transcriptomes into cell types, we performed graph-based Louvain clustering in Seurat (v 3.1.5)^13^ with different resolution depending on the analysis (overview of all cells and integration: resolution = 0.5; sporozoite and ookinete development: resolution = 0.6; sporozoite activation: resolution = 0.4). Transcriptomes were visualized using UMAP nonlinear dimensionality reduction in either Scater (v1.16.0)^66^ with n_neighbors=5, min_dist=1, spread=3 (global analysis, Fig. 1) or Seurat with n.neighbors=5, min.dist=2, and spread=3 (integration, Fig. 2) or default parameters (all other analyses).

#### Gene graph and clustering

To create the kNN gene graph, cells from cluster 0 and 1 (Fig. 1c, sporozoite clusters) were downsampled to 100 cells each in order to get a more even representation across cell types. Feature selection was performed on the expression matrix of these 623 transcripts to identify the top 2000 biologically relevant genes using selectFeature() in scmap (v 1.10.0)^25^. Transcripts were further filtered by removing those with fewer than 200 total reads across the 623 cells, resulting in 1797 genes that were used to generate the gene graph. The counts matrix was normalized by gene by dividing the mean counts for each gene and log scaling in order to reduce the amount in which clusters were driven by total expression level. The kNN graph was made using this gene normalized expression matrix with the nearest neighbors subpackage in python’s scikitlearn (v 0.2.22.post1) with n_neighbors=6 and a manhattan distance metric^67^. Spectral clustering was then performed on the kNN graph using the SpectralClustering subpackage of scikitlearn (v 0.2.22.post1)^68,69^. The kNN graph was then rendered in Gephi with force atlas 2 layout algorithm in linlog mode with scale of 0.2 and gravity of 0.8^70^. To test for functional class enrichment in each gene cluster, a Gene Ontology (GO) analysis was conducted using the GO analysis tool on PlasmoDB. Pearson’s correlations between ApiAP2 TFs expressed across the data set and each gene cluster were computed using the cor function in R.

#### Integration with *P. berghei* data

To identify conserved and divergent patterns of expression in *Plasmodium* transmission stages, we compared our *P. falciparum* data to *P. berghei* data from^8,71^. To do this, we first subsetted similar stages across the two data sets. (*P. falciparum*: gametocytes, ookinetes, and all sporozoite collections; *P. berghei*: gametocytes, ookinetes, and gland and injected sporozoites). We then built expression matrices for each data set based on one-to-one orthologs as described in^8^. Using these orthologous expression matrices, we integrated the two data sets using Seurat’s integration function by first identifying anchors with the FindIntegrationAnchors() and then using IntegrateData()^13^. Cells were visualized and clustered based on the integrated expression values as described above. Analysis of conserved markers and DE between species was then performed on each cluster using the FindConservedMarkers() and FindMarkers() functions in Seurat^13^. This procedure was also performed for asexual stage parasites from^6,8^ and compared with *P. falciparum* strain-specific expression patterns from^27^. Strain-specific expression within each matched cluster was identified using FindMarkers() in Seurat. Clusters were matched across data sets using the scmapCell() function in scmap (v 1.10.0)^25^ with the integrated smart-seq2 data as the reference index and the multi-strain data as the query data set. Each cell was given a cluster and/or stage assignment based on the top-matched cell from the reference index. Scmap was also used to compare *P. berghei* and *P. falciparum* transmission stages using the *P. berghei* orthologous expression matrix as the reference index.

#### Pseudotime

Ookinetes and sporozoites were ordered along two independent developmental trajectories using Slingshot (v 1.4.0)^72^. In brief, Seurat clusters and UMAP cell embeddings were used as input to build a minimum spanning tree to infer the order of the clusters. Slingshot then fits principal curves to estimate pseudotime values for each cell. In both cases, Slingshot identified a single trajectory that described the progression of the cells along developmental pseudotime. To identify genes differentially expressed as a function of pseudotime, we used the differentialGeneTest function in Monocle 2 (v 2.14.0)^73^. DE genes were clustered hierarchically using the pheatmap package with a Pearson correlation distance measure (clustering_distance_rows = “correlation”). Enrichment of GO terms in each pseudotime cluster was tested using the GO analysis tool on PlasmoDB. To assess whether developmentally regulated genes showed variable expression between individual cells of the same developmental stage, we identified HVGs using M3Drop (v 1.12.0), which exploits the observation that dropout-rates are correlated with expression level and identifies outliers of this null expectation as highly variable and of potential biological importance^74^. For sporozoites, ooSpz, hlSpz, sgSpz, and injSpz cells were analyzed separately. For the analysis of HVGs in ookinete cells, we first used a general linear model to regress out the effect of pseudotime, as previously described^8^. HVGs with a *q* value < 0.05 were then intersected with the pseudotime-regulated gene subset.

#### Correlation analysis

Pearson and Spearman correlations were computed using the cor or correlatePairs functions in R. Statistical significance was assessed using the cor.test function.

#### Motif discovery

To find short DNA motifs enriched in the gene clusters from Fig. 1d, the 1000 bp upstream of the start codon of each gene in each cluster were analyzed with DREME^75^ (MEME suite 5.3.3). The negative data set included the equivalent 1000 bp region of all genes outside the cluster being analyzed. Motifs were considered enriched if the *e* value was lower than 0.05. Tomtom^76^ (MEME suite 5.3.3) was used to compare the top-scoring motif of each cluster with transcription *cis*-regulatory elements described in *P. falciparum*^77–80^.

#### Differential abundance testing

To test for batch effects, we performed differential abundance analysis on single-cell salivary gland sporozoite transcriptomes derived from three independent experimental batches using the miloR package (v 0.1.0)^81^ to define cell neighborhoods on a kNN graph (*k* = 20, *d* = 10) and test for differential abundance in each neighborhood using a negative binomial general linear model framework.

#### Comparison with sporozoite bulk transcriptomes

Sporozoite single cells were assigned one of two stages (ooSpz or sgSpz) based on gene expression correlations between scRNA-seq (this study) and bulk^30^ transcriptomes. Pearson and Spearman correlations were computed using the corr package in R. To determine whether a transcript was likely to be translationally repressed (TR), we intersected scRNA-seq pseudotime gene expression profiles with the published proteomes of ooSpz and sgSpz^30^. Sporozoite transcripts that were expressed in our data set but absent in the proteome of the same stage were labeled as TR (Supplementary Data 1). Alternatively, sporozoite transcriptomes were assigned to one of two transcriptional activation states (salivary gland or activated) by computing gene expression correlations between this and *P. vivax* bulk RNA-seq data^46^.

### Reporting summary

Further information on research design is available in the Nature Research Reporting Summary linked to this article.

## Supplementary information

Supplementary Information

Peer Review File

Description of Additional Supplementary Files

Supplementary Data 1

Supplementary Data 2

Supplementary Data 3

Supplementary Data 4

Supplementary Data 5

Supplementary Movie 1

Reporting Summary

## Data Availability

Raw sequence data are available from the European Nucleotide Archive (accession number ERP124136). Expression matrices and supporting files are available on Zenodo at 10.5281/zenodo.4719664 and data are searchable via the MCA website: www.malariacellatlas.org. *P. berghei* Malaria Cell Atlas data are available at 10.5281/zenodo.2843883.

## Code availability

The code used for data analysis is available at 10.5281/zenodo.4719664.
