# Supplementary material for: A single-cell atlas of Plasmodium falciparum transmission through the mosquito
Source: Nat Commun. 2021 May 27;12:3196. doi: 10.1038/s41467-021-23434-z (PMC8159942; doi:10.1038/s41467-021-23434-z)
Supplement: Supplementary file 1 — Supplementary Information [file 41467_2021_23434_MOESM1_ESM.pdf]

SUPPLEMENTARY INFORMATION

A single-cell atlas of *Plasmodium falciparum* transmission through the mosquito

Eliana Real, Virginia M. Howick, Farah A. Dahalan, Kathrin Witmer, Juliana Cudini, Clare Andradi-Brown, Joshua Blight, Mira S. Davidson, Sunil Kumar Dogga, Adam J. Reid, Jake Baum, Mara K. N. Lawniczak

Supplementary Figures 1-19

Supplementary Tables 1-2

Supplementary References

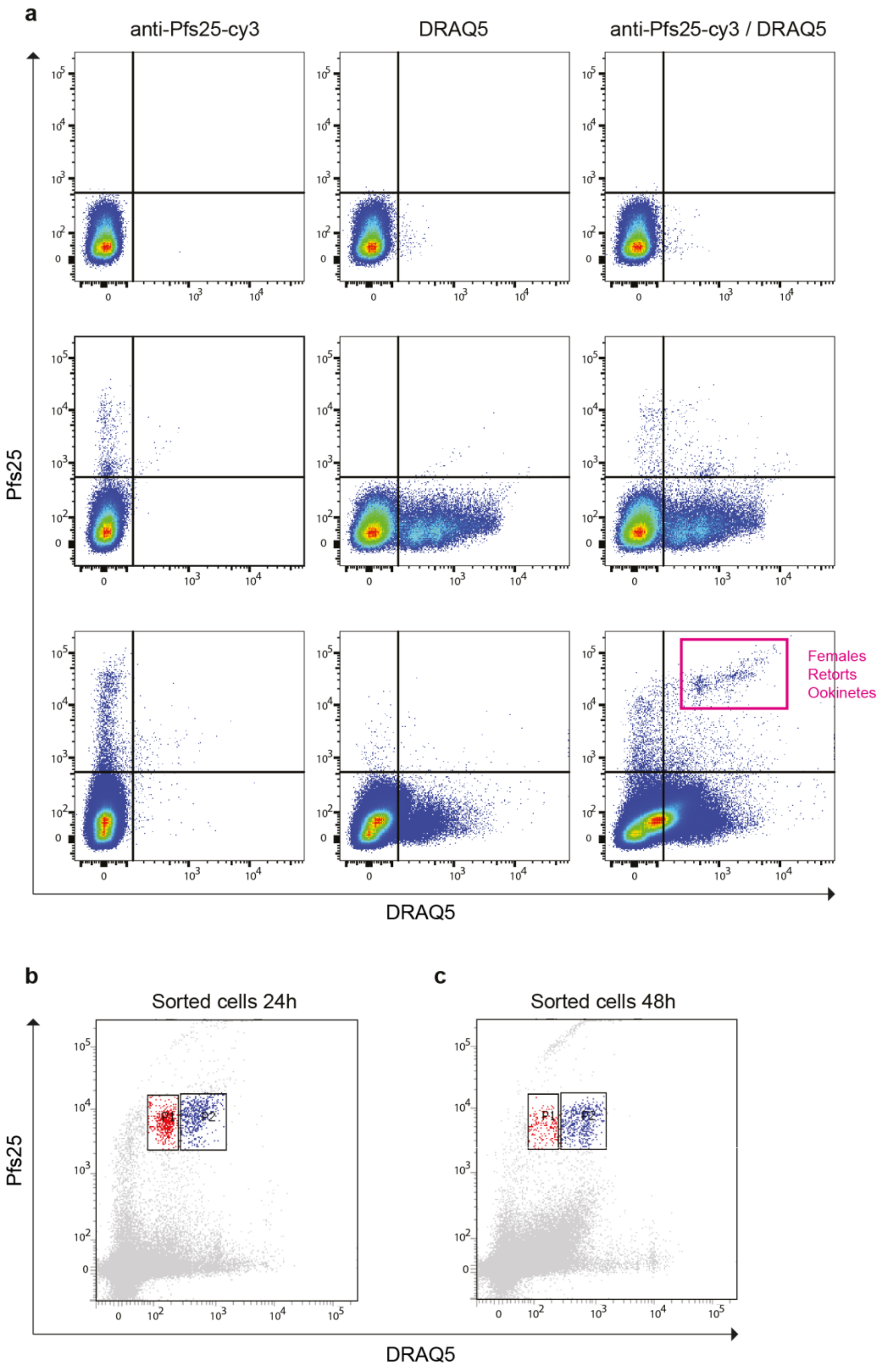

Figure S1. **Retrieval of *P. falciparum* ookinetes. a** Pilot flow cytometry experiment using the DNA-marker DRAQ5 and the anti-Pfs25 antibody coupled to cy3, to identify *P. falciparum* ookinetes. Uninfected RBCs and activated stage V gametocytes were used as controls. Double-positive cells, which comprise activated female gametes, retorts and ookinetes, were collected from *Anopheles stephensi* midgut boli 24 hours after an infectious blood feed. **b, c**

FACS scatter plots showing the two populations (P1 and P2) that were collected for the 24 h (**b**) and 48 h (**c**) post-feed timepoints.

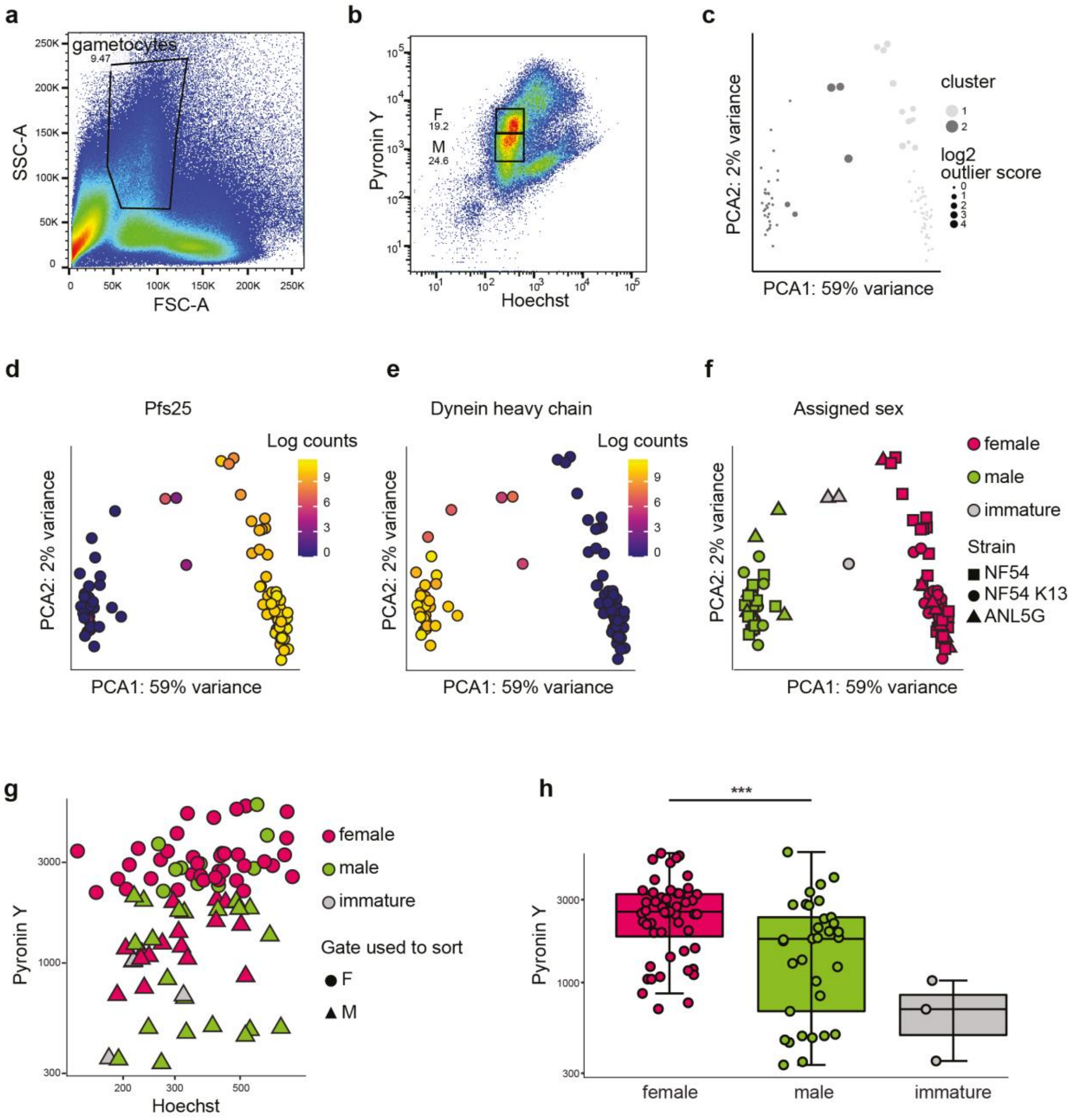

Figure S2. **Use of DNA/RNA double staining to sort *P. falciparum* gametocytes by sex. a** Scatter plot showing Forward (FSC) and Side-scatter (SSC) of an unpurified 15-day-old gametocyte culture. **b** Scatter plot showing the population gated in (a) stained with DNA (Hoechst) and RNA (Pyronin Y) dyes. Gates around the two cell populations used for single cell sorting are shown. Gates are labelled F for females and M for males. **c** Cluster analysis of the sorted gametocyte populations. Each dot represents one gametocyte. Dot sizes represent the outlier score of the cluster assignments. **d, e** Principal component analysis (PCA) of gametocyte single cell transcriptomes, highlighting expression of Pfs25, a female-specific marker (**d**), and dynein heavy chain protein (PF3D7_0905300), a male-specific marker (**e**). **f** PCA showing the assigned sex for each cell, based on data from (c-e). The different shapes represent the three different parasite lines sorted, two deriving from the

canonical lab strain NF54 background, with a third deriving from a recently culture-adapted Cambodian field isolate, APL5G. Gametocytes from the three parasite lines group together by sex on the PCA, reflecting the similar transcriptional profiles between strains. **g** RNA and DNA content in sorted gametocytes. Cells are coloured by their assigned sex and shaped according to the gate used for sorting. RNA and DNA stains are indicated in the y- and x-axis, respectively. **h** Box plots summarising the findings from (g). Female gametocytes are significantly enriched by pyronin Y stain compared to male gametocytes (Welch's t-test, *p* value = 4.873e-05), indicated by asterisks. Boxes indicate the median and interquartile ranges, while whiskers denote the data range within 1.5x of the interquartile range.

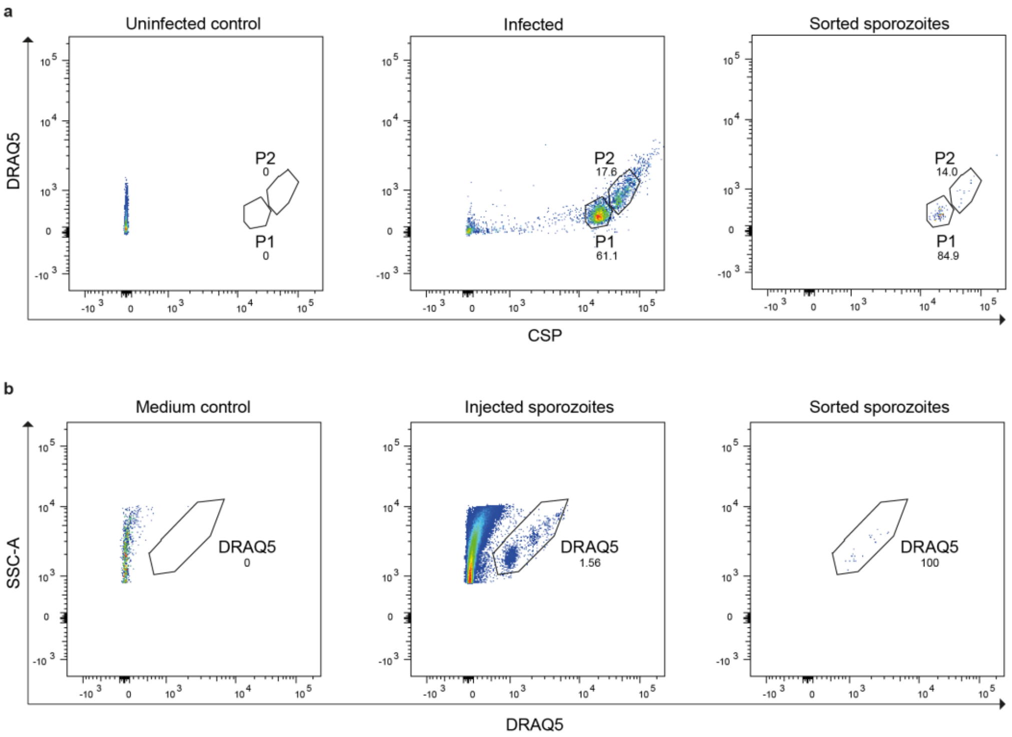

Figure S3. **Retrieval of sporozoites. a** Gating strategy for sorting sporozoite cells. Partially purified cells (see Methods) were stained with the DNA-stain DRAQ5 and anti-CSP antibody to sort sporozoites from mosquito debris. 80% of all cells were collected from gate P1, where sporozoites formed a compact population. **b** Sporozoites released through a mosquito bite were sorted based on the DRAQ5 signal alone, as no mosquito debris was detected in the sample either visually or by qPCR.

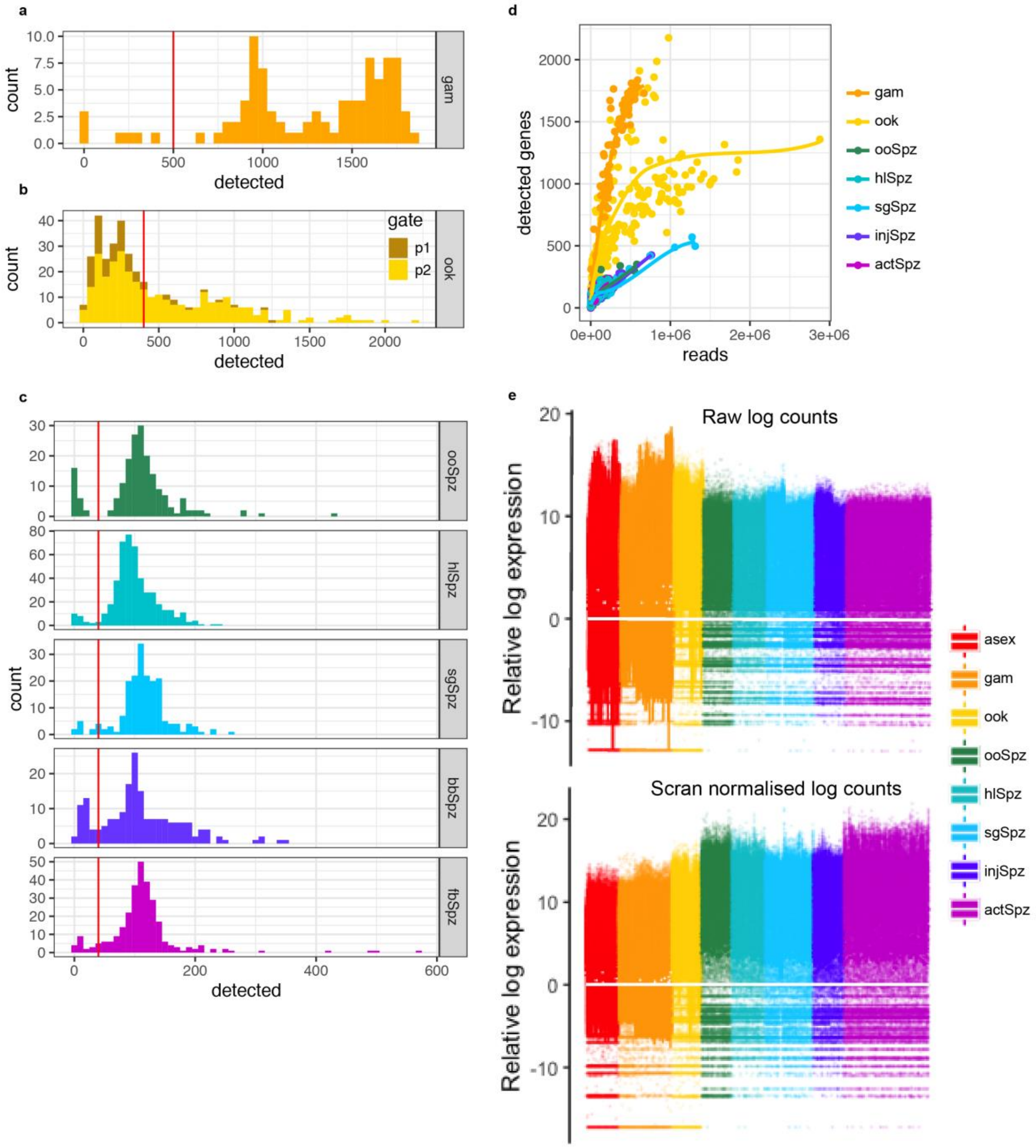

Figure S4. **Quality control and normalisation.** Filtering of cells was based on the distribution of the number of genes per cell within each parasite stage. **a** The distribution of genes detected in gametocytes. Cells with fewer than 500 genes were removed. **b** The distribution of genes detected in ookinetes. Cells with fewer than 400 genes were removed. Cells are colored according to the sorting gate (Supplementary Fig. 1). The majority of cells from p1, which had lower DNA content, were excluded based on this threshold, supporting the hypothesis that these may be dead or dying unfertilised macrogametes. **c** The distribution of genes detected in sporozoites (faceted by collection). Cells with fewer than 40 genes were removed. **d** The number of reads per cell versus the number of genes detected varied depending on parasite stage. Gametocytes and ookinetes with fewer than 10000 reads were

removed. Sporozoites with fewer than 5000 reads were removed. **e** Cell-wise relative log expression plots with raw log counts (top panel) and scran normalised log counts. Normalisation somewhat smoothed the heterogeneity seen between parasite stages.

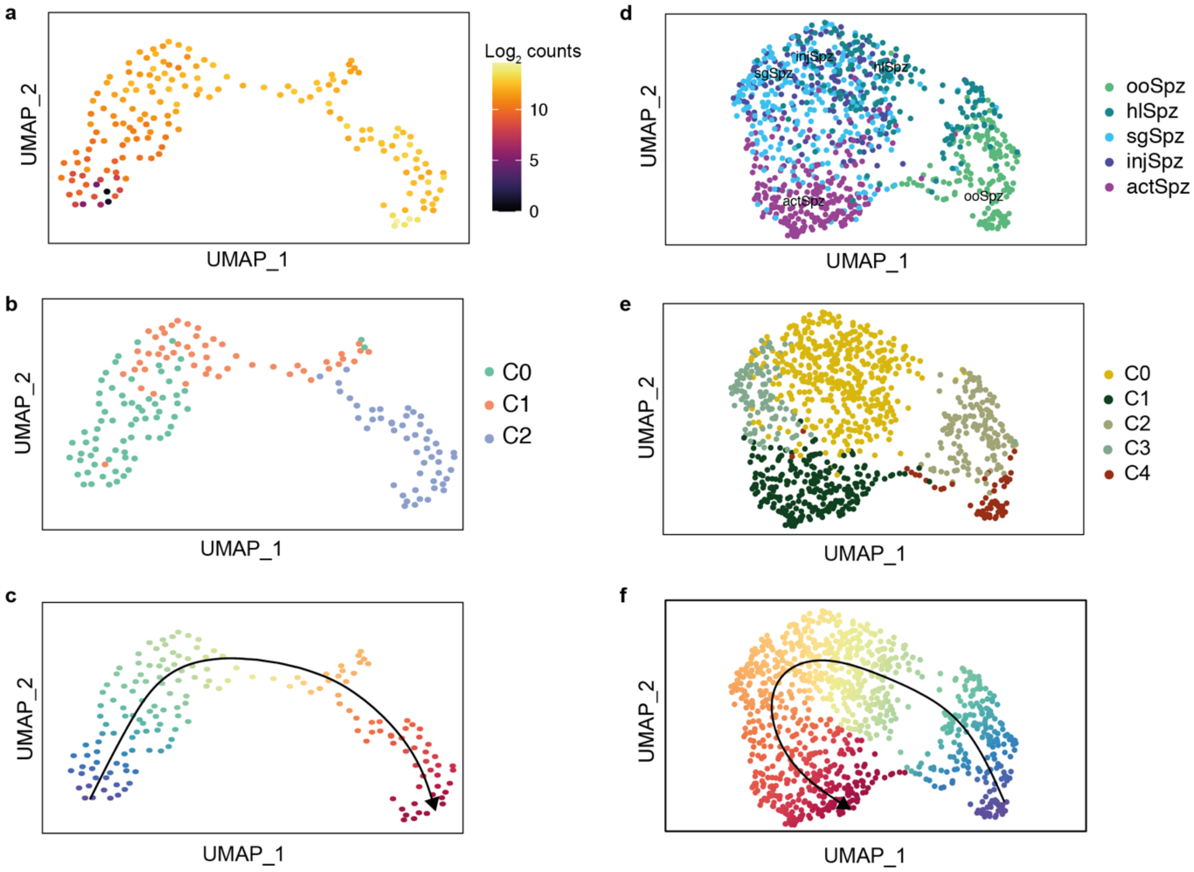

Figure S5. **Pseudotime ordering of asexual and sporozoite transcriptomes for gene graph. a-c** UMAP of asexual stage transcriptomes from [1] coloured by expression of the late-stage marker MSP1 (PF3D7_0930300)[2] (**a**), Seurat cluster assignment (**b**), and pseudotime progression (**c**). **d-f** UMAP representations of all sporozoite transcriptomes in the data set coloured by sorted stage (**d**), Seurat cluster assignment (**e**), and pseudotime (**f**). The lines in (c) and (f) represent the pseudotime trajectory. Pseudotime values were calculated using the Seurat clusters in (b) and (e) as input to Slingshot and then used to order cells in Fig. 1E by their developmental progression.

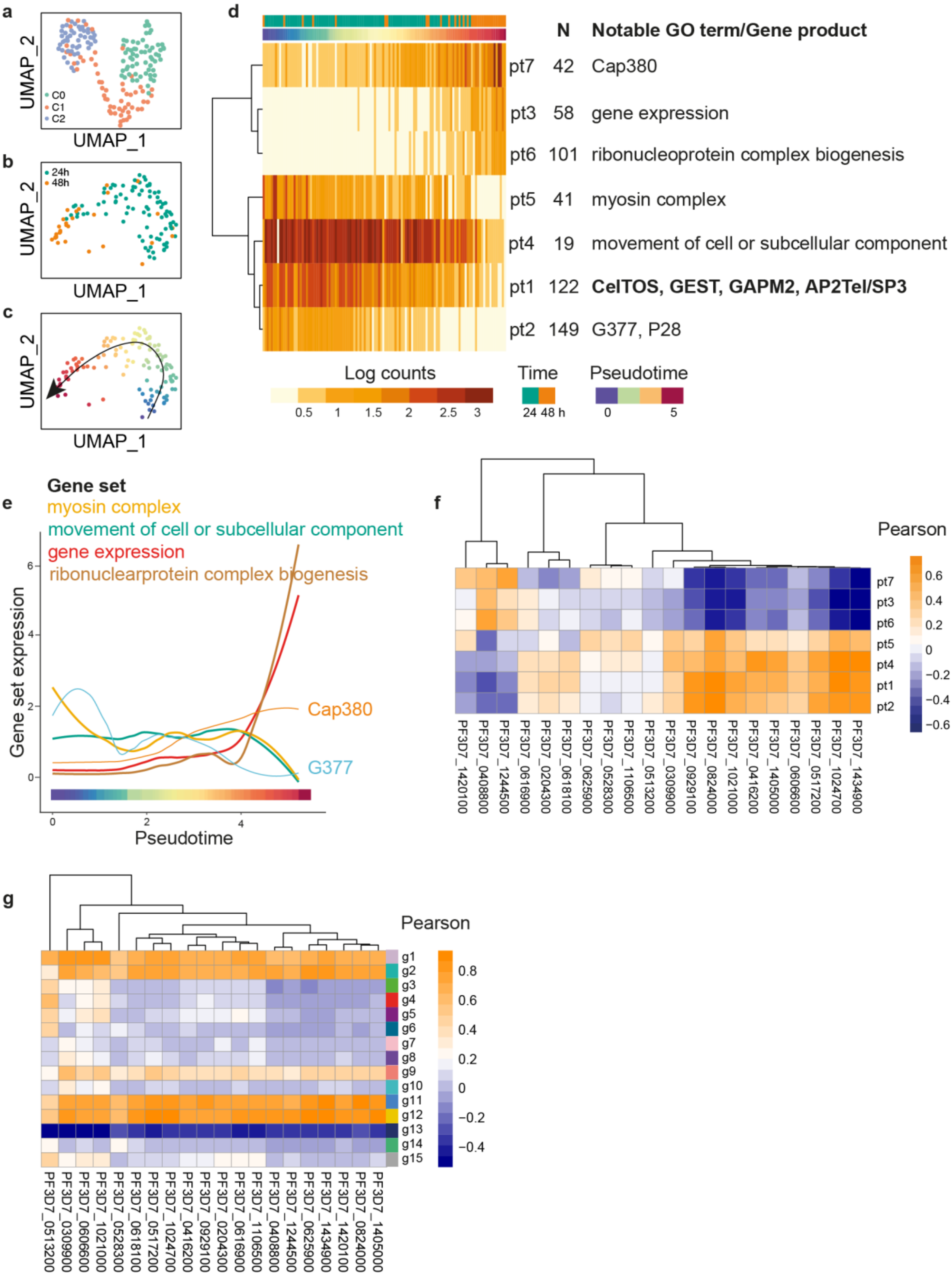
a
UMAP_2
UMAP_1
C0
C1
C2
b
24h
48h
c
d
N
Notable GO term/Gene product
pt7 42 Cap380
pt3 58 gene expression
pt6 101 ribonucleoprotein complex biogenesis
pt5 41 myosin complex
pt4 19 movement of cell or subcellular component
pt1 122 CelTOS, GEST, GAPM2, AP2Tel/SP3
pt2 149 G377, P28
Log counts
0.5 1 1.5 2 2.5 3
Time
24 48 h
Pseudotime
0 5
e
Gene set
myosin complex
movement of cell or subcellular component
gene expression
ribonuclearprotein complex biogenesis
Cap380
G377
Gene set expression
Pseudotime
f
Pearson
pt7
pt3
pt6
pt5
pt4
pt1
pt2
0.6
0.4
0.2
0
−0.2
−0.4
−0.6
PF3D7_1420100
PF3D7_0408800
PF3D7_1244500
PF3D7_0616900
PF3D7_0204300
PF3D7_0618100
PF3D7_0625900
PF3D7_0528300
PF3D7_1106500
PF3D7_0513200
PF3D7_0309900
PF3D7_0929100
PF3D7_0824000
PF3D7_1021000
PF3D7_0416200
PF3D7_1405000
PF3D7_0606600
PF3D7_0517200
PF3D7_1024700
PF3D7_1434900
g
Pearson
g1
g2
g3
g4
g5
g6
g7
g8
g9
g10
g11
g12
g13
g14
g15
0.8
0.6
0.4
0.2
0
−0.2
−0.4
PF3D7_0513200
PF3D7_0309900
PF3D7_0606600
PF3D7_1021000
PF3D7_0528300
PF3D7_0618100
PF3D7_0517200
PF3D7_1024700
PF3D7_0416200
PF3D7_0929100
PF3D7_0204300
PF3D7_0616900
PF3D7_1106500
PF3D7_0408800
PF3D7_1244500
PF3D7_0625900
PF3D7_1434900
PF3D7_1420100
PF3D7_0824000
PF3D7_1405000

Figure S6. **Ookinete development. a** UMAP representation of single-cell transcriptomes from ookinetes coloured by cluster assignment. Cluster 2, which was composed primarily of cells from the 48-hour collection, was excluded from further analysis as it lacked expression of known maker genes. We hypothesize these cells could represent dying 'ghost' ookinetes, which are at high prevalence at this time point[3]. **b, c** UMAP plots excluding cells from cluster 2 colored by time post blood meal (**b**) and developmental pseudotime (**c**). We identified 533 DE genes over pseudotime (q-value < 0.001) that clustered into 7 modules (pt1-pt7) with different expression dynamics. **d** Heatmap showing the mean expression of each cluster of DE genes over pseudotime with cells ordered by their pseudotime value. The number of genes (N) and top GO terms in each cluster are shown. Bonferroni adjusted *p* values for this analysis are shown in Supplementary Data 4. Examples of highly variable genes between individual ookinetes are indicated in bold next to the cluster to which they belong. HVGs were determined using a general linear model to regress out the effect of pseudotime and M3Drop with FDR < 0.05. Heterogeneity in the expression of genes associated with cell traversal (CelTOS) and motility (GAPM2) suggests phenotypic heterogeneity with respect to midgut colonisation. **e** Expression of sets of functionally related genes in each cluster over pseudotime. **f** Heatmap of Pearson's correlations between the expression of 20 species-conserved (*P. berghei* and *P. falciparum*) genes of unknown function and gene clusters from (d). Patterns of correlated expression can help assign roles for genes that lack functional annotation. For instance, expression of PF3D7_1434900, PF3D7_1024700, and PF3D7_0416200 is highly correlated with that of genes involved in cell movement, suggesting a possible role in ookinete motility. **g** Pearson's correlations between the expression of the genes in (f) and the gene clusters from Fig. 1D. All but 2 genes show high correlation with clusters specific to (g11, g12), or associated with (g1, g2) ookinetes, but not with asexual (g3, g4, g5, g6, g14, g15) or sporozoite (g13) stages.

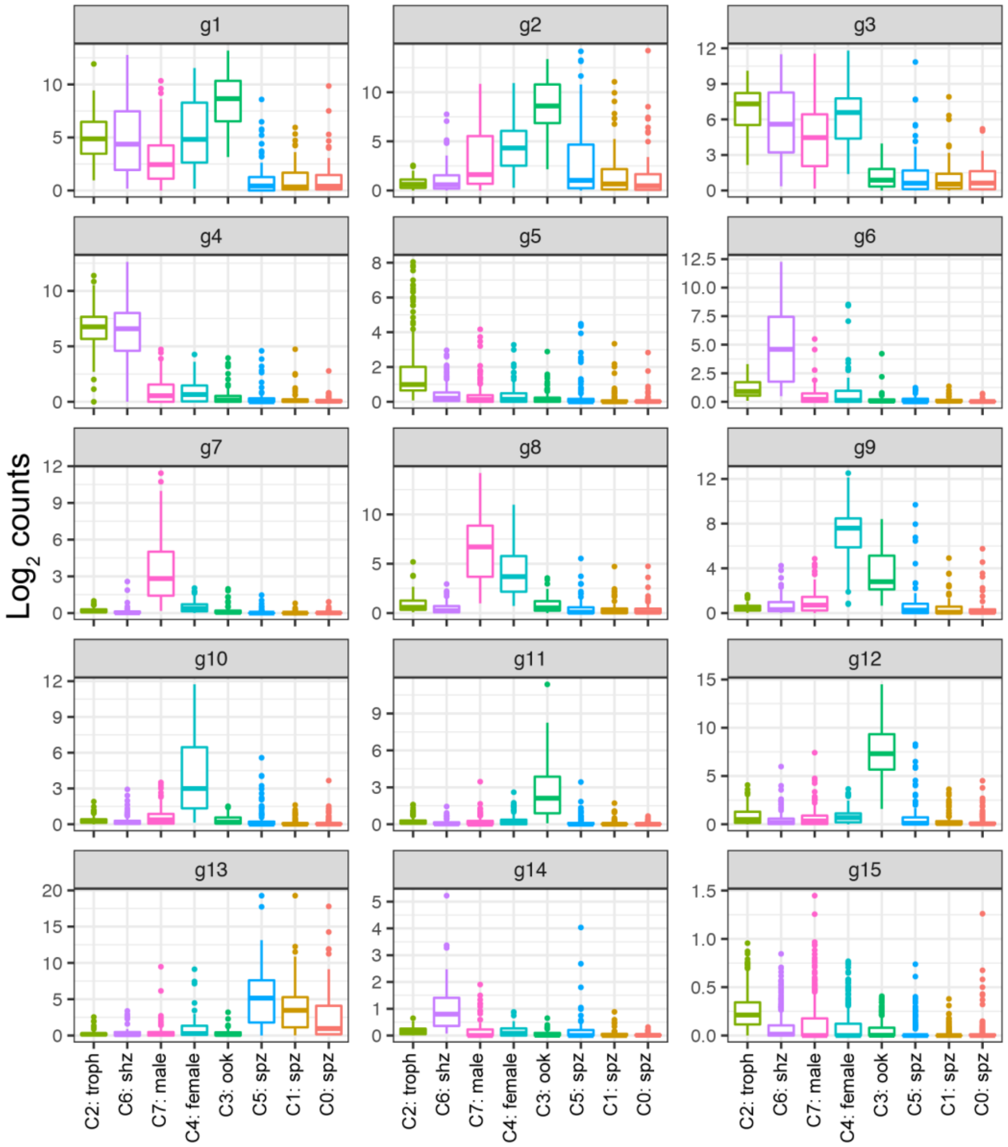

Figure S7. **Expression of each gene cluster by cell-type.** The mean expression of all genes in each cluster (Fig. 1D) by parasite cell cluster (Fig. 1C). The lower and upper hinges of the box-plot correspond to the first and third quantiles. Overall level and cell type-specific expression patterns varies across the clusters.

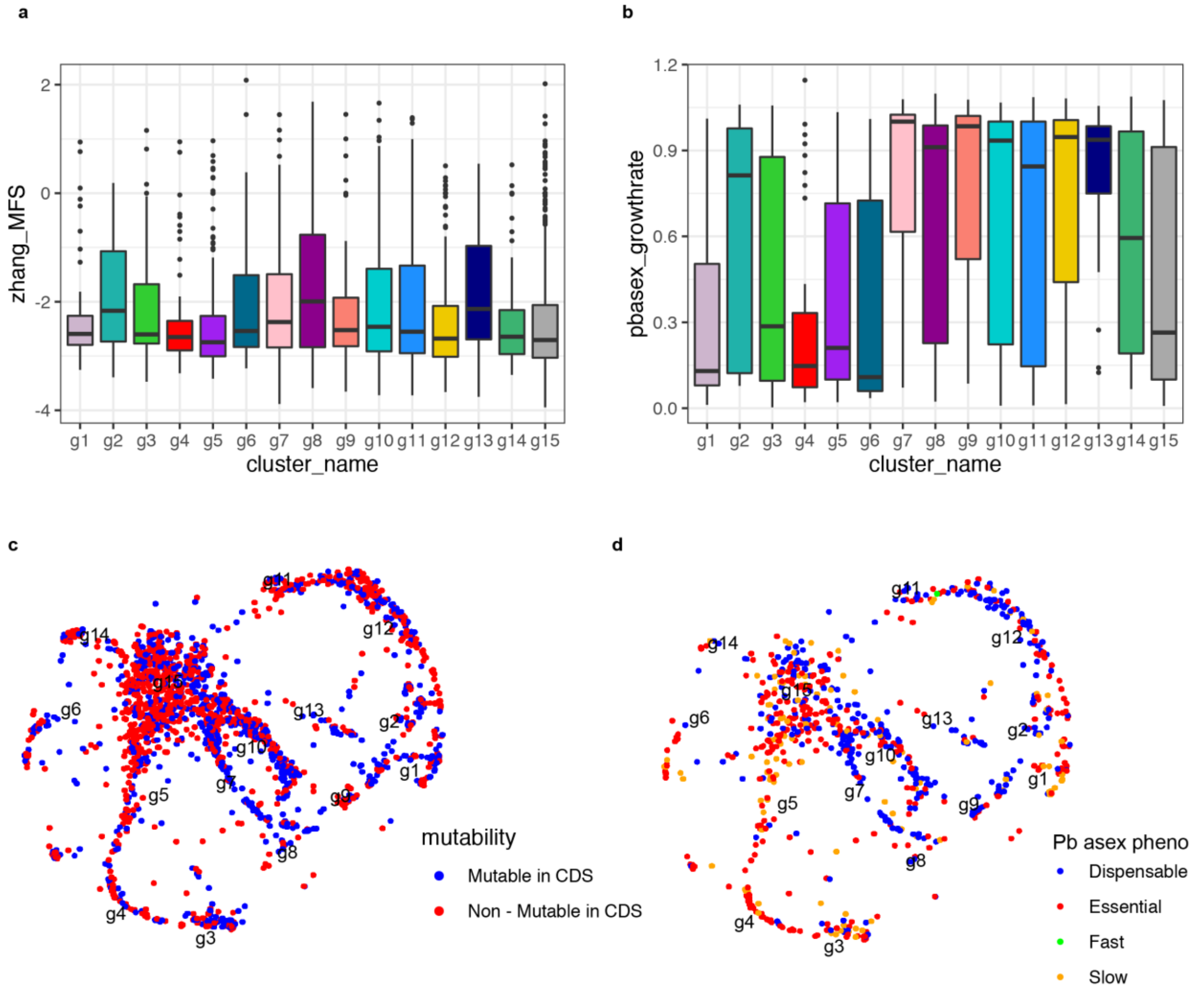

Figure S8. **Essentiality across the gene graph. a-d** Variation in essentiality across gene clusters when compared to[4] and[5]. Clusters varied in their mutagenesis fitness score (MFS, ANOVA: $p = 5.80 \times 10^{-6}$) (**a**) and growth-rate of the orthologous genes in *P. berghei* (ANOVA: $p < 2.2 \times 10^{-16}$) (**b**). We found that g7 and g8 (highly expressed in male gametocytes) as well as g13 (highly expressed in sporozoites) were enriched for mutable genes, whereas g5 (highly expressed in asexual blood stages) was enriched for non-mutable genes from[4] (Fisher's Exact Test (FET), one-sided; FDR < 0.05, Supplementary Table 2) (**c**)**.** Additionally, g7 (male), g9/g10 (female), g12 (ookinete) and g13 (sporozoite) were enriched for dispensable orthologous *P. berghei* genes and g4 (asexual blood stages) was enriched for essential orthologous genes identified in [5] (FET, one-sided: FDR < 0.05, Supplementary Table 2) (**d**)**.** In the boxplots, boxes indicate the median and interquartile ranges, while whiskers denote the data range within 1.5x of the interquartile range. Dots represent the outliers beyond this range.

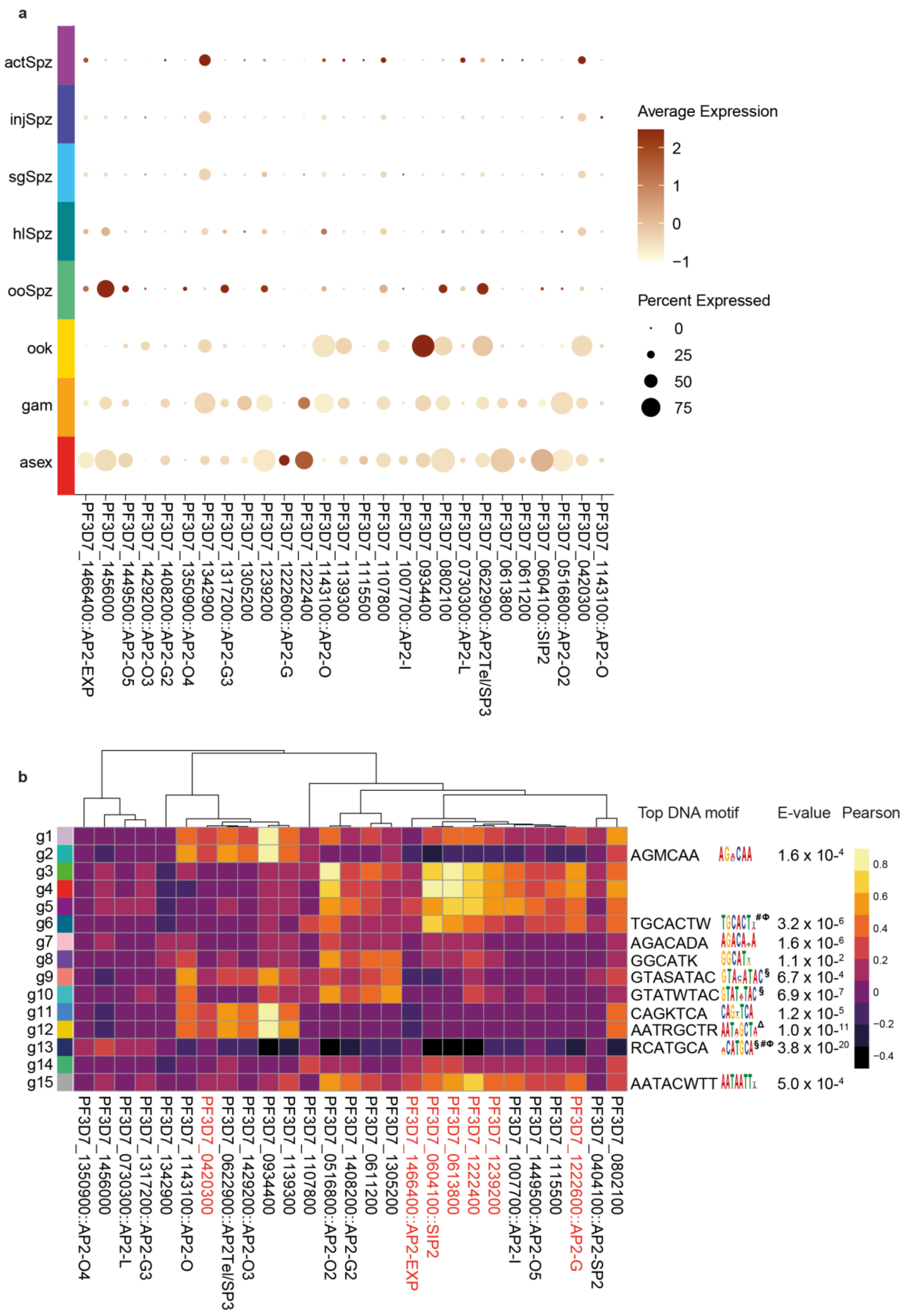
a
actSpz
injSpz
sgSpz
hlSpz
ooSpz
ook
gam
asex
Average Expression
2
1
0
−1
Percent Expressed
0
25
50
75
PF3D7_1466400::AP2-EXP
PF3D7_1456000
PF3D7_1449500::AP2-O5
PF3D7_1429200::AP2-O3
PF3D7_1408200::AP2-G2
PF3D7_1350900::AP2-O4
PF3D7_1342900
PF3D7_1317200::AP2-G3
PF3D7_1305200
PF3D7_1239200
PF3D7_1222600::AP2-G
PF3D7_1222400
PF3D7_1143100::AP2-O
PF3D7_1139300
PF3D7_1115500
PF3D7_1107800
PF3D7_1007700::AP2-I
PF3D7_093440O
PF3D7_0802100
PF3D7_0730300::AP2-L
PF3D7_0622900::AP2Tel/SP3
PF3D7_0613800
PF3D7_0611200
PF3D7_0604100::SIP2
PF3D7_0516800::AP2-O2
PF3D7_0420300
PF3D7_1143100::AP2-O
b
Top DNA motif
E-value
Pearson
g1
g2
g3
g4
g5
g6
g7
g8
g9
g10
g11
g12
g13
g14
g15
AGMCAA 1.6 x 10-4
TGCACTW 3.2 x 10-6
AGACADA 1.6 x 10-6
GGCATK 1.1 x 10-2
GTASATAC 6.7 x 10-4
GTATWTAC 6.9 x 10-7
CAGKTCA 1.2 x 10-5
AATRGCTR 1.0 x 10-11
RCATGCA 3.8 x 10-20
AATACWTT 5.0 x 10-4
0.8
0.6
0.4
0.2
0
−0.2
−0.4
PF3D7_1350900::AP2-O4
PF3D7_1456000
PF3D7_0730300::AP2-L
PF3D7_1317200::AP2-G3
PF3D7_1342900
PF3D7_1143100::AP2-O
PF3D7_0420300
PF3D7_0622900::AP2Tel/SP3
PF3D7_1429200::AP2-O3
PF3D7_0934400
PF3D7_1139300
PF3D7_1107800
PF3D7_0516800::AP2-O2
PF3D7_1408200::AP2-G2
PF3D7_0611200
PF3D7_1305200
PF3D7_1466400::AP2-EXP
PF3D7_0604100::SIP2
PF3D7_0613800
PF3D7_1222400
PF3D7_1239200
PF3D7_1007700::AP2-I
PF3D7_1449500::AP2-O5
PF3D7_1115500
PF3D7_1222600::AP2-G
PF3D7_0404100::AP2-SP2
PF3D7_0802100

Figure S9. **Expression of ApiAP2 transcription factors across the life cycle of *P. falciparum*.** **a** Dot plot showing the average expression (colour-coded) and proportion of cells of each stage (dot size) that express each of the 26 ApiAP2 TFs detected in the data set. **b** Pearson's correlations (colour-coded) between ApiAP2 gene expression and the gene clusters from Fig. 1D. The seven ApiAp2 TFs highlighted in red are ApiAP2s for which BDP1 association with their promoter regions has been demonstrated[6], suggesting they are subject to epigenetic regulation. Most are strongly correlated with asexual and sexual stages of the life cycle. DNA motifs enriched within 1 Kb upstream of the start codon of genes in each cluster are shown (E-value < 0.05), except when no statistically significant enrichment was found (Supplementary Data 5). Motifs identified in previous studies are marked with §[7], #[8], Φ[9], and △[10] and show the expected correspondence with the life cycle stage they were linked with. The top motif in cluster g12 corresponds to the AP2-O binding site identified in △[10], while the most significant motifs in clusters g6 and g13 match the binding sites for SIP2 and AP2-EXP, respectively[9]. The former has been shown to bind to subtelomeric chromatin in *P. falciparum* and has been implicated in the silencing of upsB *var* genes[11], which are represented in cluster g6. Our analysis shows that expression of SIP2 is highly correlated with that cluster, which is consistent with SIP2's putative role in *var* gene regulation.

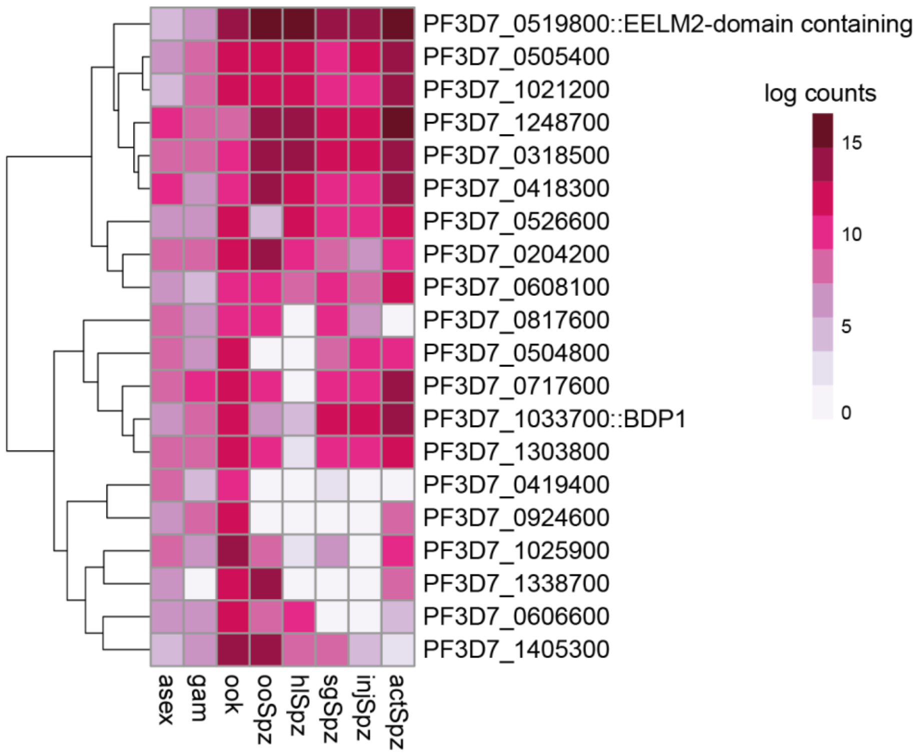

Figure S10. **Expression patterns of unannotated genes from cluster g1.** Average per stage expression of BDP1, PF3D7_0519800 (EELM2-domain containing) and 18 genes from cluster g1 lacking functional annotation. Genes with BDP1- or EELM2-like patterns of expression can be observed.

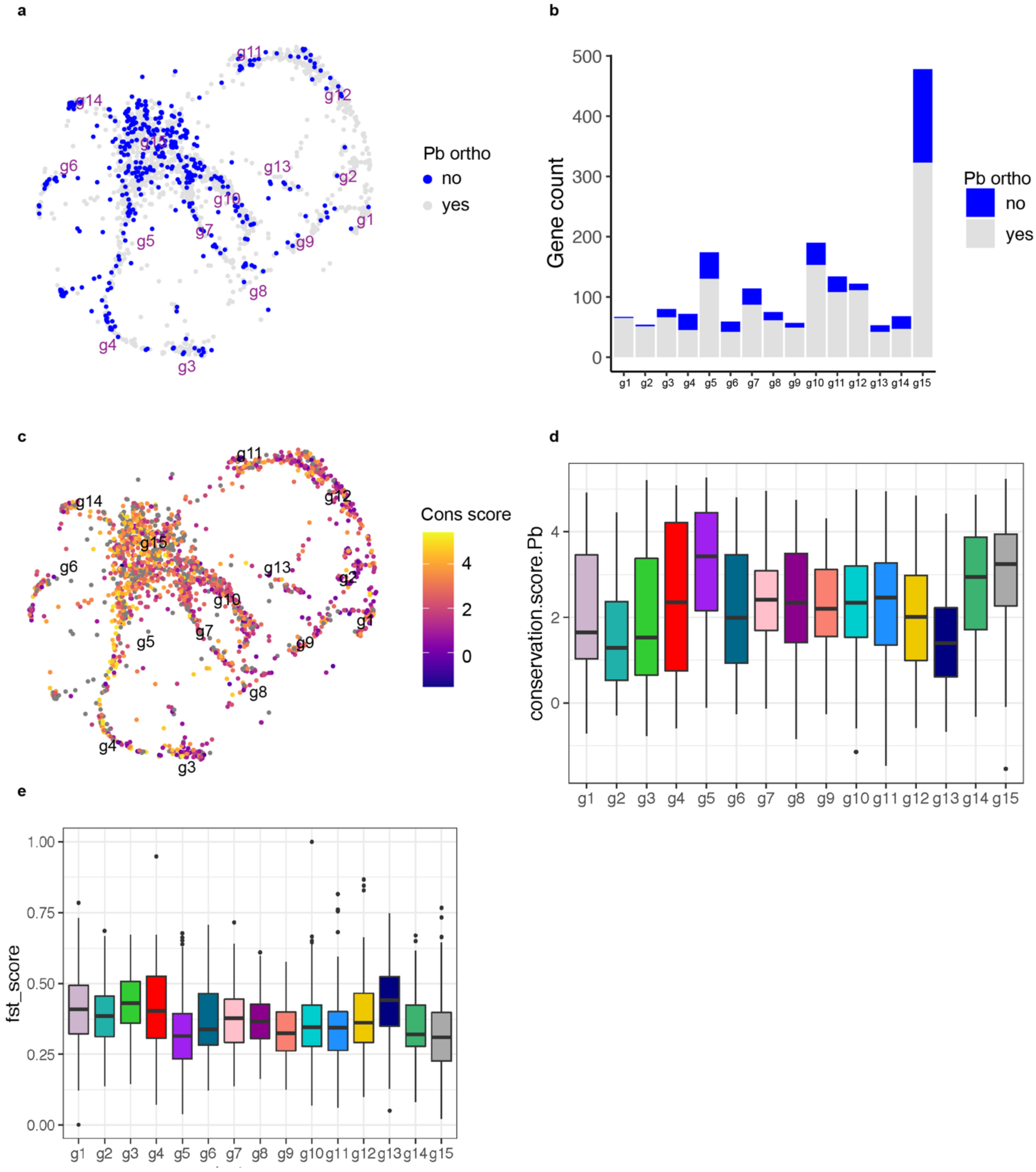

Figure S11. **Signatures of selection across the kNN gene graph. a** The kNN gene graph with non-orthologous genes highlighted in blue. **b** A barplot of gene counts for each gene cluster (g1-g15) coloured by orthology. Clusters g4 and g15 were significantly enriched for genes that have no orthologs with *P. berghei*, and clusters g1, g2, g12 had more genes with one-to-one orthologs than expected by chance (FET, two-sided; FDR < 0.05, Supplementary Table 2). **c** Amino acid conservation score between *P. falciparum* and *P. berghei* based on mean substitution score for all amino acids in the protein[1,12]. **d** A boxplot of conservation score by gene cluster. There was significant variation in conservation score across the clusters (ANOVA, $p < 2.2 \times 10^{-16}$) with clusters g2 and g13 having the lowest median conservation score. These clusters are also enriched for genes involved in host-parasite interactions. **e** A boxplot of the global FST score from[13] by gene cluster. There was significant

variation in FST score across the clusters (ANOVA, $p < 2.2 \times 10^{-16}$). g13 had the highest median global differentiation score. The high global differentiation, in combination with enrichment in genes involved in host-parasite interactions and low amino acid conservation, suggests that genes in this cluster may be evolving in response to host factors that are species specific and vary geographically. In the boxplots, boxes indicate the median and interquartile ranges, while whiskers denote the data range within 1.5x of the interquartile range. Dots represent the outliers beyond this range.

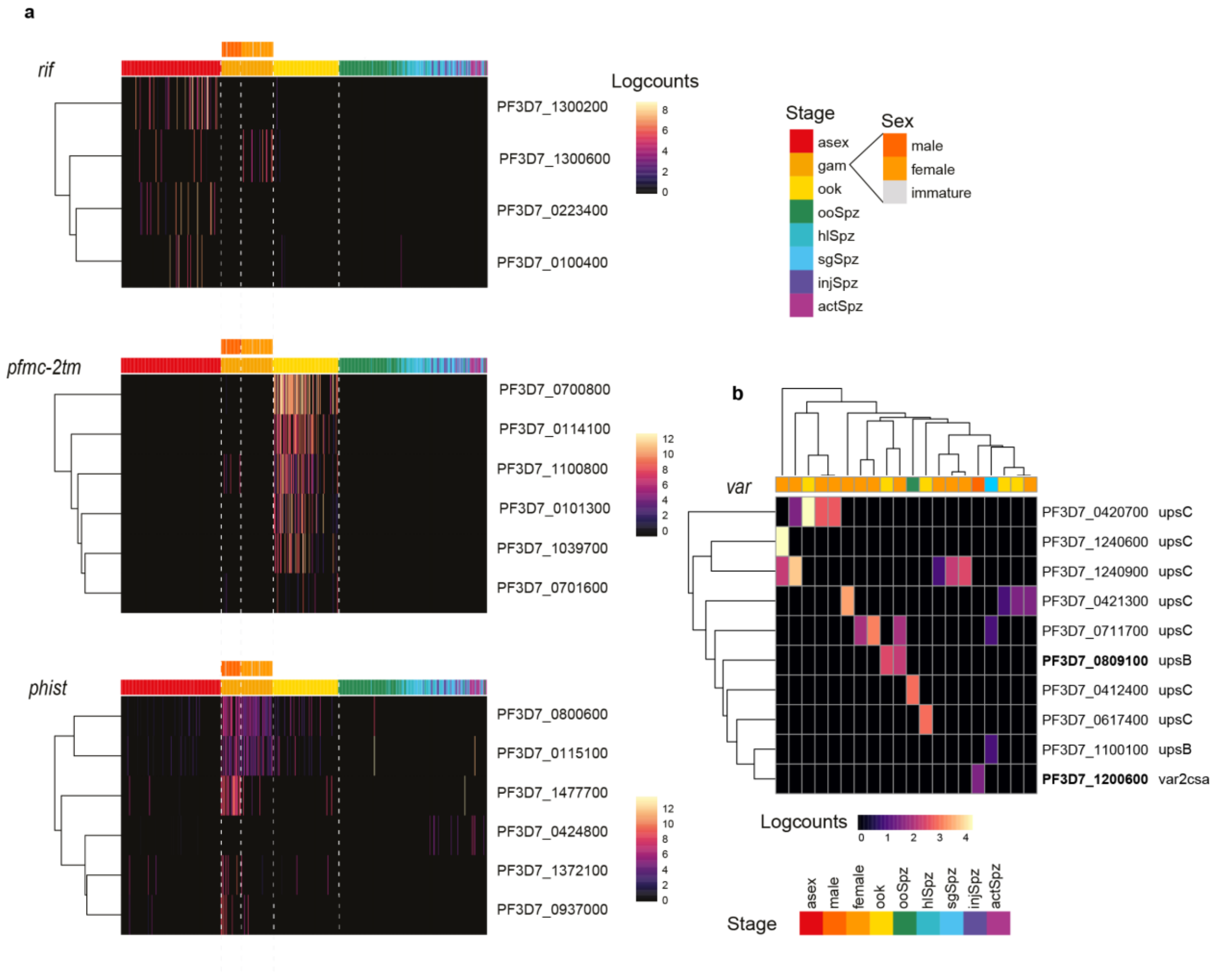

Figure S12. **Expression of *P. falciparum* multigene families across the life cycle.** **a** Heatmaps showing the expression levels of genes without 1:1 *P. berghei* orthologs across the life cycle. Only genes that belong to clonally variant multigene families[14] and expressed in at least 10 cells across the data set are shown. Ookinetes are significantly enriched for expression of six genes belonging to the PfMC-2TM family (Bonferroni adjusted p value = 1.2 $\times 10^{-05}$), which have been localised to the host-parasite interface in asexual parasite forms[15] and were not known until now to be expressed in this stage of the life cycle. Expression of PfMC-2TM family members is associated with ookinete development over pseudotime (q-value < 0.01) (Supplementary Data 1). **b** Expression levels of members of the *var* multigene family for which sense transcripts could be detected. We identified sense transcripts using split reads spanning the var intron as described in[1] and focused on these because ncRNA is expressed from the body of members of this gene family. Each column in the heatmap represents a single cell colour-coded by its life cycle stage. The type of each *var* gene type is indicated as upstream promoter sequence (ups) B/C or *var2csa*. Although we were only able to confirm sense transcription of *vars* in a small number of cells, several of the identified transcripts had been previously identified in transmission stages. PF3D7_0809100 (an upsB type *var* gene; highlighted in bold), which has been shown to be translated in *P. falciparum* NF54 sporozoites in[16] but not in[17], is detected in female gametocyte and ookinete cells. Additionally, PF3D7_1200600 (*var2csa*; highlighted in bold) which has been primarily associated with pregnancy-associated malaria and has recently been shown to be expressed in male gametocyte bulk transcriptomic data[1,18] was here detected in a single male

gametocyte. The majority of cells where we detected sense *var* transcripts displayed mutually exclusive expression (expressing a single *var* gene). However, we detected simultaneous expression of two *var* genes in four cells (female gametocytes and sporozoite), but in these cases, one or both *vars* were expressed at very low levels (fewer than 5 sense reads detected).

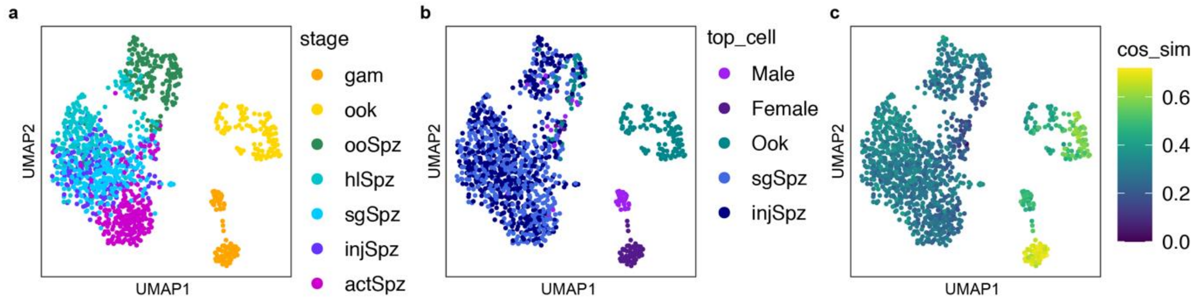

Figure S13. **scmap of *P. falciparum* transcriptomes to *P. berghei* dataset.** Scmap[19] was used to compare our data with equivalent stages from the *P. berghei* data set[20]. A reference index for *P. berghei* was built based on one-to-one orthologs and the *P. falciparum* data was mapped to it to match each cell to a *P. berghei* cell. **a-c** UMAP of *P. falciparum* cells coloured according to collection stage (**a**), stage of the matched *P. berghei* cell (**b**), and cosine similarity metric for each cell (**c**). In general, cells matched to a cell from a similar stage in the *P. berghei* dataset. Female gametocytes and ookinetes had a higher cosine similarity on average compared to sporozoites and male gametocytes, which also tend to have fewer genes per cell.

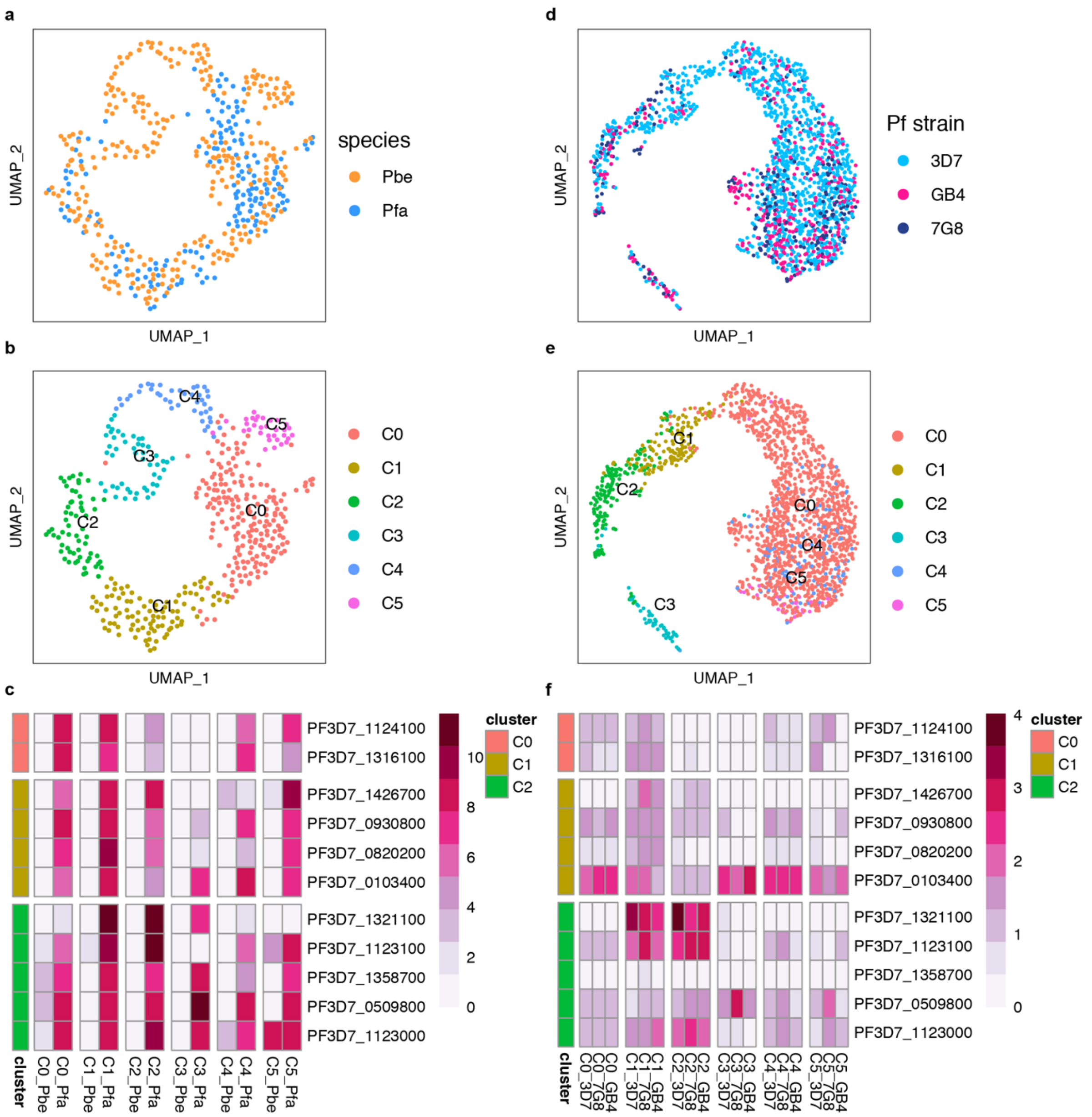

Figure S14. ***P. falciparum* specific expression patterns in asexual stage parasites.** To understand differential expression patterns between *P. berghei* and *P. falciparum* in asexual stage parasites, we integrated single-cell trophozoite and schizont data from[1,20] using one-to-one orthologs. **a, b** UMAP of integrated data coloured by species **(a)** and cluster assignment **(b)**. Clusters C0, C1, and C2 were used for differential expression analysis as they had good representation of both species. **c** Expression (mean $\log_2$ counts) of the top one-to-one orthologs that are more highly expressed in *P. falciparum* (Pfa compared to *P. berghei* (Pbe) (based on adjusted *p* value and expression in at least 70% of *P. falciparum* cells). **d-f** To understand how the observed species-specific expression could be driven by differences between genotypes within a species, we returned to asexual data collected using the 10x technology from[21] that profiled expression from diverse *P. falciparum* strains including

3D7, GB4 and 7G8 (ERP119738). Data from these three strains are shown on the UMAP coloured by strain **(d)**. Matching cell clusters from the integrated data set in (b) were identified using scmap and are shown on the UMAP in **(e)**. Expression of the species-specific transcripts across the three strains shows that they are not differentially expressed between strains **(f)**.

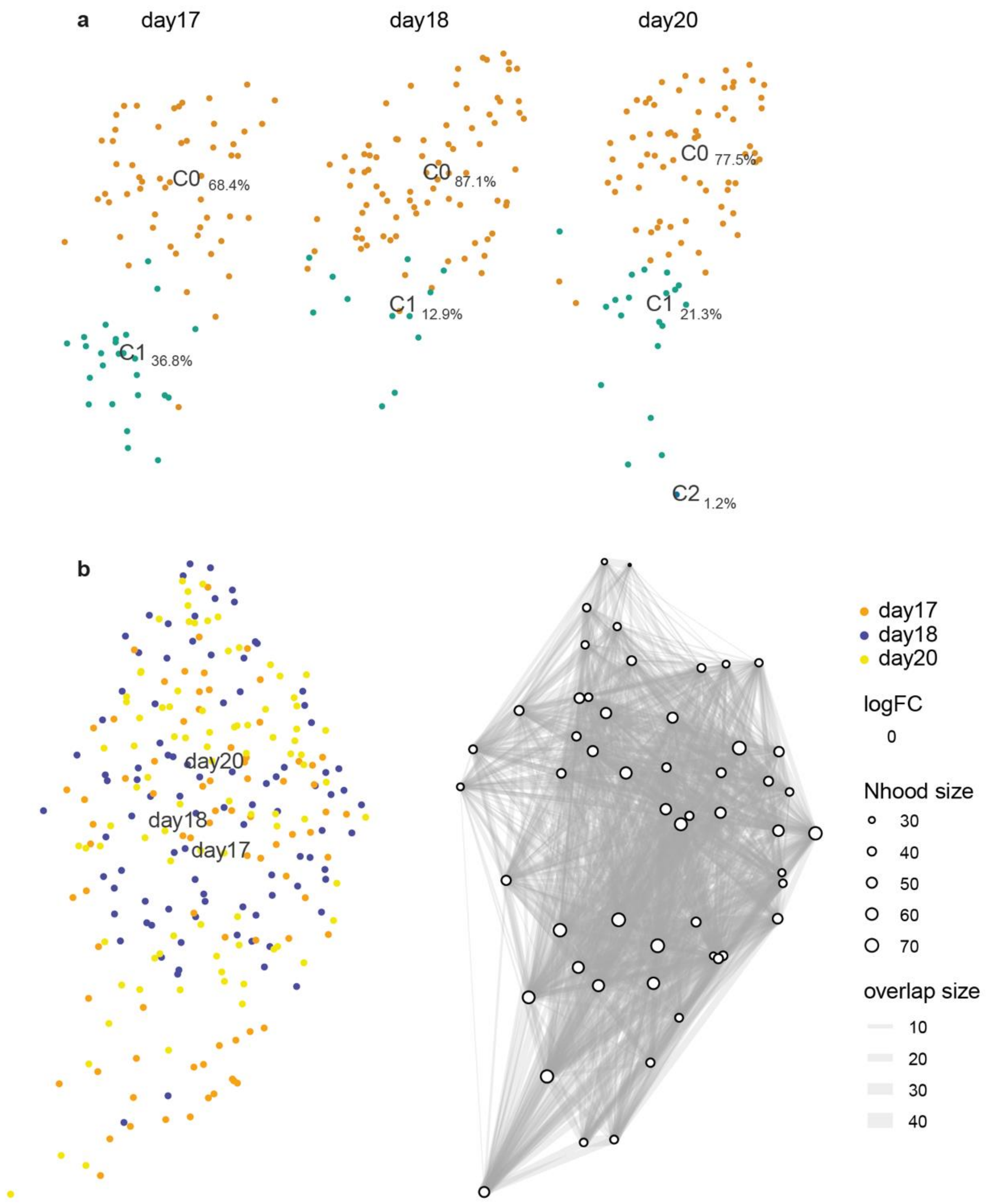

Figure S15. **Reproducibility between experimental batches. a** UMAP of salivary gland sporozoite transcriptomes from three independent experimental batches (day17, day18, day20) coloured by cluster assignment. The percentage of cells in each cluster is indicated. The distribution of cells per cluster is similar between batches. **b** UMAP embedding (left) and graph representation of neighbourhoods (right) of cells from (a). Each node represents a neighbourhood whose size (Nhood size) is proportional to the number of cells in that neighbourhood. The differential abundance (logFC) between experimental batches in the 51 neighbourhoods identified was found not to be significant (FRD 5%).

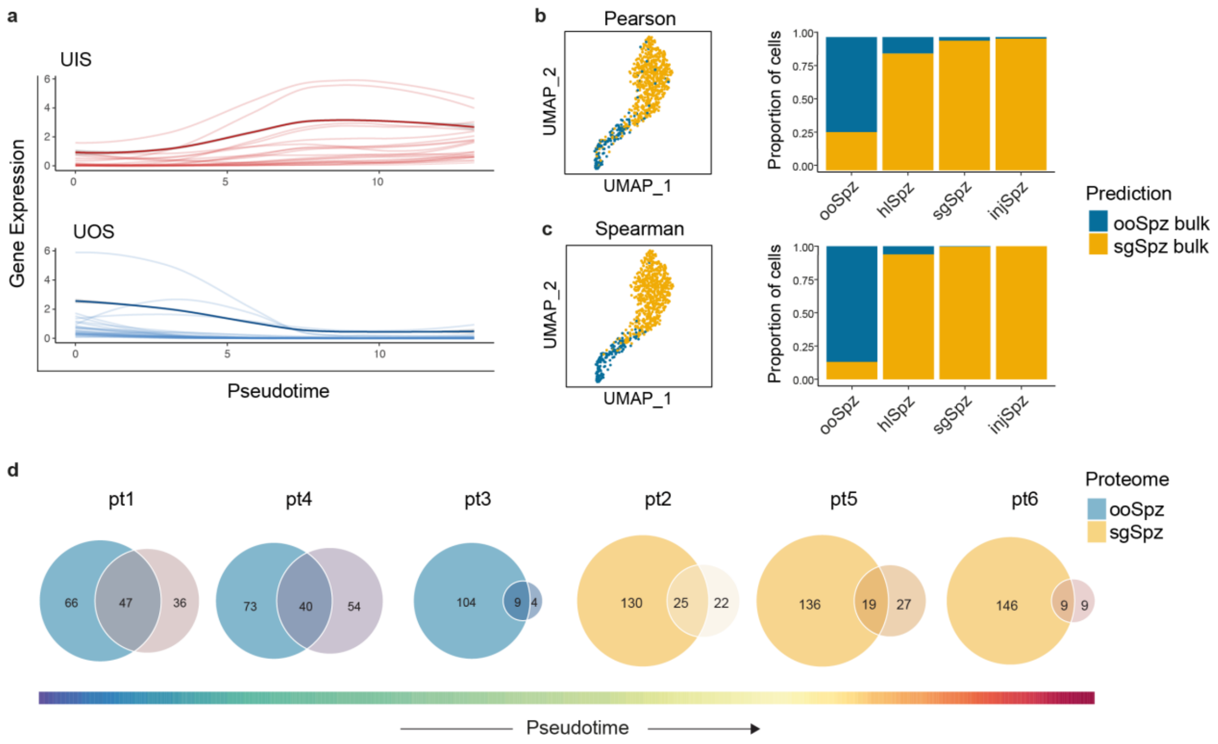

Figure S16. **Comparison of the sporozoite transcriptional programme between single-cell and bulk data sets. a** Expression dynamics of 21 UIS (top) and 29 UOS (bottom) genes identified by Lindner *et al*.[17] that show DE over pseudotime (q-val <0.0001) . Individual genes are represented by light-coloured lines and the average expression of each gene set is shown in darker colours. **b, c** Predicted sporozoite stage based on Pearson (**b**) and Spearman (**c**) correlations with available bulk data. The left and right panels show UMAP representations of single-cell transcriptomes coloured by predicted stage and the quantitative distribution of cells according to their predicted stage, respectively. Single-cell transcriptomes matched to either oocyst or salivary gland bulk data as expected (Supplementary Data 2). **d** Correspondence between mRNA and protein expression for DE genes over pseudotime. Proteins where the gene showed differential expression over pseudotime were selected from the two proteome datasets (ooSpz and sgSpz) from[17] and compared to each pseudotime gene cluster (pt 1-6) from the same stage. The Venn diagrams show the intersection of each cluster of DE genes (pt 1-6) with ooSpz or sgSpz sub-proteomes[17]. Sporozoite transcripts for which no protein products could be detected in Lindner *et al*. might be translationally repressed.

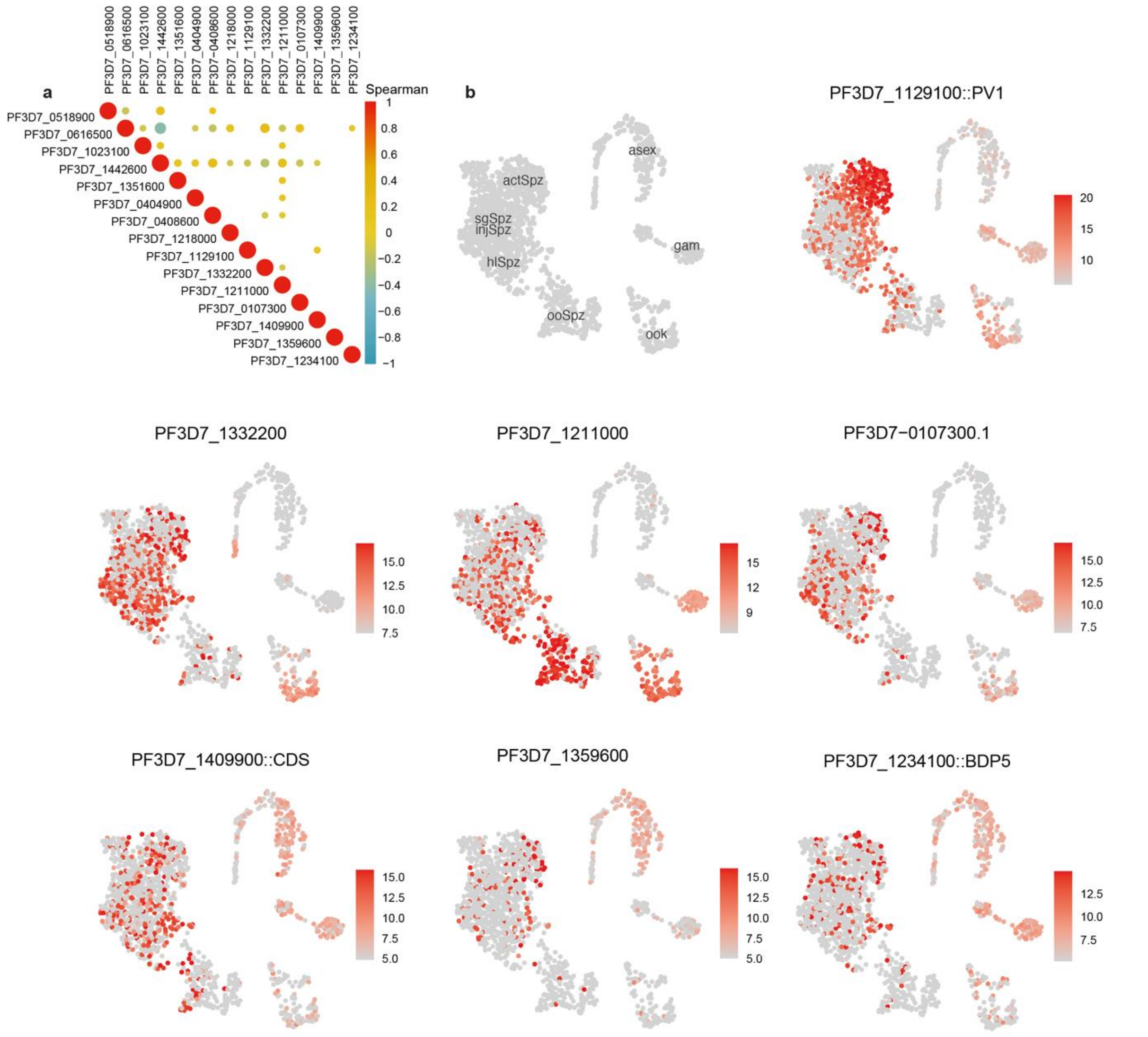

Figure S17. **Highly variable genes in sporozoites. a** Pairwise Spearman correlations between sporozoite (sgSpz, injSpz) HVGs. Significant values (FDR < 0.05) are represented by circles. The range of Spearman correlation coefficients (*rho)* measured do not indicate a strong positive or negative correlation between HVGs, implying that HVGs are not co-regulated. **b** UMAPs of single cell transcriptomes from across the parasite life cycle with the expression of HVGs in sgSpz and injSpz highlighted. Gene expression levels are colour-coded according to the scale (log counts) next to each plot. The life cycle key is shown in the first UMAP.

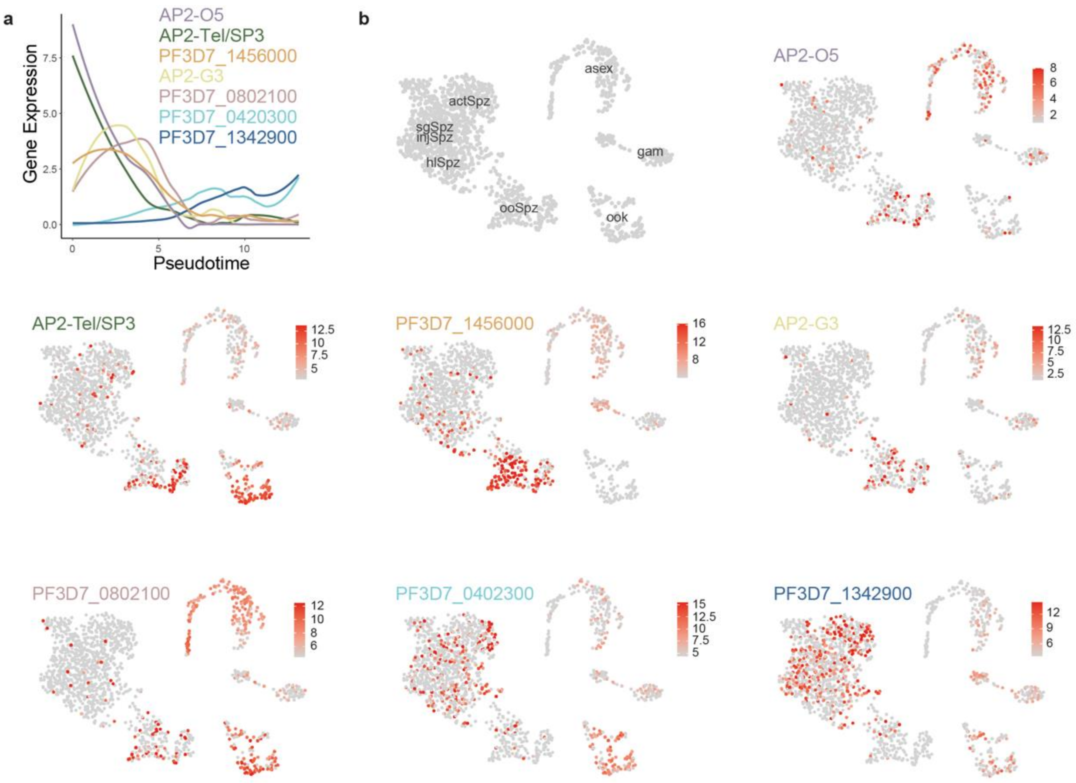

Figure S18. **ApiAP2 transcription factors associated with sporozoite development in the mosquito. a** Expression of ApiAP2 transcription factors DE along sporozoite pseudotime. **b** UMAP representations of the parasite life cycle with the expression levels of ApiAP2 TFs from (a) colour-coded according to the scale (log counts) next to each plot. Only AP2-Tel/Sp3 (PF3D7_0622900) has been previously implicated in sporozoite development in *P. berghei*[22]. AP2-G3 (PF3D7_1317200) and AP2-O5 (PF3D7_1449500) have known roles in sexual development and ookinete motility, respectively[23]. The first UMAP shows the life cycle key.

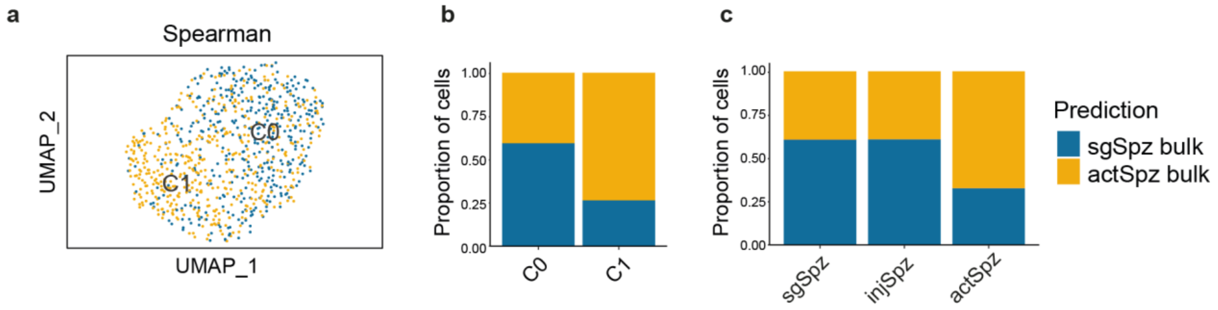

Figure S19. **Comparison of single-cell and bulk transcriptomes upon sporozoite activation. a-c** Predicted sporozoite activation status based on Spearman correlations with available bulk data[24] from *P. vivax* (Schneider's t = 0 h *versus* RPMI + 3% BSA t = 4 h). The left (**a**) and right (**b, c**) panels show a UMAP of single-cell transcriptomes coloured by predicted activation status and the quantitative distribution of cells according to their predicted activation status, respectively. Cluster assignments (C0, C1) are indicated as per Fig. 4. Single-cell transcriptomes matched to either salivary gland or activated bulk data as expected (Supplementary Data 2).

Supplementary Table 1. **Quality control of single-cell transcriptomes by isolation method.**

Stages purified	Time point	# before QC	Genes per cell cut off	Reads per cell cut off	# Passed QC	Success rate	Median genes per cell	Median reads per cell	Genes per stage (% coverage)
**Gametocyte**	Day 16	95	500	10000	88	93%	1496.5	3.31×10^5	4547 (79.60%)
**Ookinete**	24 h & 48 h	383	400	5000	165	43%	829	5.10×10^5	4088 (71.57%)
**Oocyst spz**	Day 12	190	40	5000	161	85%	125	1.07×10^5	2429 (42.52%)
**Hemolymph spz**	Day 14	190	40	5000	178	94%	126	1.08×10^5	2195 (38.43%)
**Gland spz**	Day 17, 18, 20	285	40	5000	261	92%	124	1.05×10^5	2827 (49.49%)
**Injected spz**	Day 17, 18	190	40	5000	163	86%	125	1.10×10^5	2258 (39.53%)
**Activated spz**	Day 20	475	40	5000	451	95%	108	7.72×10^4	2452 (42.93%)

Gametocyte time point is represented as days post gametocyte induction, all other time points are hours or days post infectious feed. The percent coverage was calculated as the genes detected across cells in each stage divided by the total number of genes in the genome (5712).

Supplementary Table 2. **Per cluster statistics across the knn gene graph.**

Cluster	N	Mean MFS	N mutable	Mutable FDR FET (one-sided)	N non-mutable	Non-mutable FDR FET (one-sided)	N dispensable	Dispensable FDR FET (one-sided)	N essential	Essential FDR FET (one-sided)	Mean cons score	N no orth	Orthos FDR FET (two-sided)	Mean FST score
g1	67	-2.32	26	1.00E+00	39	1.00E+00	4	1.00E+00	28	1.18E-01	2.06	1	7.88E-05	0.41
g2	54	-1.89	29	2.60E-01	23	1.00E+00	13	5.86E-01	10	1.00E+00	1.54	2	3.15E-03	0.39
g3	80	-2.12	41	3.15E-01	37	1.00E+00	10	1.00E+00	21	7.64E-01	1.98	13	3.76E-01	0.42
g4	72	-2.37	25	1.00E+00	39	1.00E+00	3	1.00E+00	30	5.23E-04	2.56	26	2.01E-02	0.41
g5	174	-2.41	47	1.00E+00	121	9.26E-03	13	1.00E+00	44	1.18E-01	3.28	43	7.38E-01	0.32
g6	59	-1.97	27	7.13E-01	30	1.00E+00	3	1.00E+00	13	2.63E-01	2.17	16	4.74E-01	0.38
g7	114	-2.00	71	8.30E-04	41	1.00E+00	31	2.99E-04	10	1.00E+00	2.41	26	1.00E+00	0.38
g8	75	-1.69	49	8.30E-04	24	1.00E+00	19	3.57E-01	10	1.00E+00	2.37	13	5.04E-01	0.37
g9	57	-2.15	28	4.44E-01	27	1.00E+00	22	8.36E-03	7	1.00E+00	2.25	7	2.77E-01	0.33
g10	190	-2.06	92	3.78E-01	91	1.00E+00	53	2.69E-03	25	1.00E+00	2.29	36	3.28E-01	0.36
g11	134	-2.05	61	7.28E-01	70	1.00E+00	28	2.17E-01	20	1.00E+00	2.33	25	4.34E-01	0.35
g12	122	-2.29	46	1.00E+00	73	1.00E+00	40	1.33E-03	14	1.00E+00	2.05	10	1.85E-04	0.39
g13	53	-1.83	33	1.60E-02	18	1.00E+00	14	8.36E-03	2	1.00E+00	1.53	10	7.96E-01	0.43
g14	68	-2.33	23	1.00E+00	41	1.00E+00	5	1.00E+00	8	7.84E-01	2.70	20	3.12E-01	0.36
g15	478	-2.30	162	1.00E+00	298	1.00E+00	55	1.00E+00	82	7.84E-01	3.12	154	1.85E-04	0.32

The number (N) of genes in each cluster as well as the number of those genes classified as mutable or non-mutable in [4], the number of those classified as dispensable or essential in [5] (see Supplementary Fig. 8), as well as the number of genes in each cluster that have no ortholog in *P. berghei* (see Supplementary Fig. 11). The FDR corrected *p* value to test for enrichment in each of these categories is also reported (Fisher's Exact Test (FET), either one-sided or two-sided). Finally, the mean values for the mutability fitness score (MFS)[4], conservation score[12], and FST score[13] are reported.

Supplementary References

1. Reid, A. J. *et al.* Single-cell RNA-seq reveals hidden transcriptional variation in malaria parasites. *Elife* **7**, (2018).
2. Das, S. *et al.* Processing of Plasmodium falciparum Merozoite Surface Protein MSP1 Activates a Spectrin-Binding Function Enabling Parasite Egress from RBCs. *Cell Host Microbe* **18**, 433–444 (2015).
3. Siciliano, G. *et al.* Critical Steps of Plasmodium falciparum Ookinete Maturation. *Front. Microbiol.* **11**, 269 (2020).
4. Zhang, M. *et al.* Uncovering the essential genes of the human malaria parasite Plasmodium falciparum by saturation mutagenesis. *Science* **360**, (2018).
5. Bushell, E. *et al.* Functional Profiling of a Plasmodium Genome Reveals an Abundance of Essential Genes. *Cell* **170**, 260–272.e8 (2017).
6. Josling, G. A. *et al.* A Plasmodium Falciparum Bromodomain Protein Regulates Invasion Gene Expression. *Cell Host Microbe* **17**, 741–751 (2015).
7. Young, J. A. *et al.* In silico discovery of transcription regulatory elements in Plasmodium falciparum. *BMC Genomics* **9**, 70 (2008).
8. Campbell, T. L., De Silva, E. K., Olszewski, K. L., Elemento, O. & Llinás, M. Identification and genome-wide prediction of DNA binding specificities for the ApiAP2 family of regulators from the malaria parasite. *PLoS Pathog.* **6**, e1001165 (2010).
9. De Silva, E. K. *et al.* Specific DNA-binding by apicomplexan AP2 transcription factors. *Proc. Natl. Acad. Sci. U. S. A.* **105**, 8393–8398 (2008).
10. Yuda, M., Iwanaga, S., Shigenobu, S., Kato, T. & Kaneko, I. Transcription factor AP2-Sp and its target genes in malarial sporozoites. *Mol. Microbiol.* **75**, 854–863 (2010).
11. Flueck, C. *et al.* A major role for the Plasmodium falciparum ApiAP2 protein PfSIP2 in chromosome end biology. *PLoS Pathog.* **6**, e1000784 (2010).
12. MalariaGEN Plasmodium falciparum Community Project. Genomic epidemiology of artemisinin resistant malaria. *Elife* **5**, (2016).

13. MalariaGEN *et al.* An open dataset of *Plasmodium falciparum* genome variation in 7,000 worldwide samples. *Wellcome Open Res.* **6**, 42 (2021).
14. Fraschka, S. A. *et al.* Comparative Heterochromatin Profiling Reveals Conserved and Unique Epigenome Signatures Linked to Adaptation and Development of Malaria Parasites. *Cell Host Microbe* **23**, 407–420.e8 (2018).
15. Tsarukyanova, I., Drazba, J. A., Fujioka, H., Yadav, S. P. & Sam-Yellowe, T. Y. Proteins of the Plasmodium falciparum two transmembrane Maurer's cleft protein family, PfMC-2TM, and the 130 kDa Maurer's cleft protein define different domains of the infected erythrocyte intramembranous network. *Parasitol. Res.* **104**, 875 (2009).
16. Zanghì, G. *et al.* A Specific PfEMP1 Is Expressed in P. falciparum Sporozoites and Plays a Role in Hepatocyte Infection. *Cell Rep.* **22**, 2951–2963 (2018).
17. Lindner, S. E. *et al.* Transcriptomics and proteomics reveal two waves of translational repression during the maturation of malaria parasite sporozoites. *Nature Communications* vol. 10 (2019).
18. Lasonder, E. *et al.* Integrated transcriptomic and proteomic analyses of P. falciparum gametocytes: molecular insight into sex-specific processes and translational repression. *Nucleic Acids Res.* **44**, 6087–6101 (2016).
19. Kiselev, V. Y., Yiu, A. & Hemberg, M. scmap: projection of single-cell RNA-seq data across data sets. *Nat. Methods* **15**, 359–362 (2018).
20. Howick, V. M. *et al.* The Malaria Cell Atlas: Single parasite transcriptomes across the complete Plasmodium life cycle. *Science* **365**, (2019).
21. Heaton, H. *et al.* Souporcell: robust clustering of single-cell RNA-seq data by genotype without reference genotypes. *Nature Methods* vol. 17 615–620 (2020).
22. Modrzynska, K. *et al.* A Knockout Screen of ApiAP2 Genes Reveals Networks of Interacting Transcriptional Regulators Controlling the Plasmodium Life Cycle. *Cell Host Microbe* **21**, 11–22 (2017).
23. Zhang, C. *et al.* Systematic CRISPR-Cas9-Mediated Modifications of Plasmodium yoelii ApiAP2 Genes Reveal Functional Insights into Parasite Development. *MBio* **8**, (2017).

24. Roth, A. *et al.* Unraveling the Plasmodium vivax sporozoite transcriptional journey from mosquito vector to human host. *Scientific Reports* vol. 8 (2018).
