# Supplementary material for: A single-cell atlas of Plasmodium falciparum transmission through the mosquito
Source: Nat Commun. 2021 May 27;12:3196. doi: 10.1038/s41467-021-23434-z (PMC8159942; doi:10.1038/s41467-021-23434-z)
Supplement: Supplementary file 3 — Description of Additional Supplementary Files [file 41467_2021_23434_MOESM3_ESM.pdf]

Description of Additional Supplementary Files

File Name: Supplementary Data 1
Description: **Summary statistics for each gene across the dataset.** Includes knn graph cluster assignment, differential gene expression analysis, analysis of conserved stage markers, highly variable genes, pseudotime association, and comparisons with bulk RNAseq.

File Name: Supplementary Data 2
Description: **Summary statistics for each cell across the dataset.** Includes cell metadata, QC metrics, cluster assignments, pseudotime ordering, and correlation with bulk RNAseq datasets.

File Name: Supplementary Data 3
Description: **Expression matrix.**

File Name: Supplementary Data 4
Description: **GO term enrichment analysis.** Significantly enriched GO terms across the knn graph and pseudotime gene clusters. GO terms for which Benjamini adjusted p value < 0.05 are shown.

File Name: Supplementary Data 5
Description: - **Promotor motif enrichment analysis.** DNA motifs significantly enriched within 1 Kb upstream of the start codon of genes in each cluster across the knn graph are provided along with E-values.

File Name: Supplementary Movie 1
Description: **MSP1 expression in egressing sporozoites.** 12- day oocyst immunostained with anti-CSP (green), anti-MSP1 (red), and Hoechst (blue). The image captures the egress of sporozoites (CSP positive) from an oocyst. Released sporozoites express MSP1 on their surface. Scale bar, 10 µm.
