# Supplementary material for: A single-cell atlas of Plasmodium falciparum transmission through the mosquito
Source: Nat Commun. 2021 May 27;12:3196. doi: 10.1038/s41467-021-23434-z (PMC8159942; doi:10.1038/s41467-021-23434-z)
Supplement: Supplementary file 10 — Reporting Summary [file 41467_2021_23434_MOESM10_ESM.pdf]

nature research

Corresponding author(s): Jake Baum, Mara Lawniczak

Last updated by author(s): Apr 22, 2021

Reporting Summary

Nature Research wishes to improve the reproducibility of the work that we publish. This form provides structure for consistency and transparency in reporting. For further information on Nature Research policies, see our Editorial Policies and the Editorial Policy Checklist.

Statistics

For all statistical analyses, confirm that the following items are present in the figure legend, table legend, main text, or Methods section.

n/a	Confirmed	
☐	☒	The exact sample size (*n*) for each experimental group/condition, given as a discrete number and unit of measurement
☒	☐	A statement on whether measurements were taken from distinct samples or whether the same sample was measured repeatedly
☐	☒	The statistical test(s) used AND whether they are one- or two-sided<br>*Only common tests should be described solely by name; describe more complex techniques in the Methods section.*
☐	☒	A description of all covariates tested
☐	☒	A description of any assumptions or corrections, such as tests of normality and adjustment for multiple comparisons
☐	☒	A full description of the statistical parameters including central tendency (e.g. means) or other basic estimates (e.g. regression coefficient) AND variation (e.g. standard deviation) or associated estimates of uncertainty (e.g. confidence intervals)
☐	☒	For null hypothesis testing, the test statistic (e.g. F, t, r) with confidence intervals, effect sizes, degrees of freedom and P value noted<br>*Give P values as exact values whenever suitable.*
☒	☐	For Bayesian analysis, information on the choice of priors and Markov chain Monte Carlo settings
☒	☐	For hierarchical and complex designs, identification of the appropriate level for tests and full reporting of outcomes
☒	☐	Estimates of effect sizes (e.g. Cohen's d, Pearson's r), indicating how they were calculated

Our web collection on statistics for biologists contains articles on many of the points above.

Software and code

Policy information about availability of computer code

Data collection	Cell sorting was performed on a BD FACSAria III with BD FACSDiva software (version 8.0). Images were acquired on a Leica SP8 confocal microscope equipped with LAS X software.
Data analysis	The following software was used for data analysis:<br><br>Analysis of FACS data was done using FlowJo (version 10.7.1) software.<br><br>Image quantification was performed with Fiji (version 2.1.0/1.53c).<br><br>Transcriptome mapping and generation of counts matrix: cutadapt (v 1.18), HISAT2 (v 2.1.0), Subread package (v 2.0.0)<br><br>Downstream expression analysis was performed in R (v 4.0.0) with the following packages: scran (v 1.16.0), Seurat (v 3.1.5), Scater (v1.16.0), scmap (v 1.10.0), M3drop (1.12.0), Monocle (2.14.0), MiloR (0.1.0), Slingshot (1.4.0)<br><br>Motif enrichment and analysis was performed in DREME and Tomtom (MEME Suite 5.3.3)<br><br>The gene graph was built using the scikitlearn package (v 0.2.22.post1) in python (v 3.8.2).<br><br>All R and python packages used to analyze the data are detailed in Methods.

For manuscripts utilizing custom algorithms or software that are central to the research but not yet described in published literature, software must be made available to editors and reviewers. We strongly encourage code deposition in a community repository (e.g. GitHub). See the Nature Research guidelines for submitting code & software for further information.

Data

Policy information about availability of data

All manuscripts must include a data availability statement. This statement should provide the following information, where applicable:

- Accession codes, unique identifiers, or web links for publicly available datasets
- A list of figures that have associated raw data
- A description of any restrictions on data availability

Raw sequence data are available from the European Nucleotide Archive (accession number ERP124136). Expression matrices and supporting files are available on Github at https://github.com/vhowick/pf_moz_stage_atlas and data are searchable via the MCA website: www.malariacellatlas.org.

Data was mapped to the P. falcipcarum v3 genome available at https://www.sanger.ac.uk/resources/downloads/protozoa/

GO analysis used the PlasmoDB database at https://plasmodb.org/

Field-specific reporting

Please select the one below that is the best fit for your research. If you are not sure, read the appropriate sections before making your selection.

☒ Life sciences ☐ Behavioural & social sciences ☐ Ecological, evolutionary & environmental sciences

For a reference copy of the document with all sections, see nature.com/documents/nr-reporting-summary-flat.pdf

Life sciences study design

All studies must disclose on these points even when the disclosure is negative.

Sample size	No statistical methods were used to predetermine sample size. We aimed to profile approximately 100 cells per parasite stage based on previous data from P. berghei that showed that this was a sufficient sample size to identify differentially expressed and highly variable patterns of expression that we were interested in. The sample size is specified in Supplementary Table S1.
Data exclusions	No datasets were excluded from the study. Quality control removed a subset of cells and was determined based on the distribution of the number of genes and reads per cell for each parasite stage as indicated in Supplementary Table 1 and Supplementary Figure 4.
Replication	The number of cells for each life cycle stage analyzed by sc-RNAseq is indicated in Supplementary Table 1. Three replicate samples were taken from the salivary gland sporozoites, and no significant batch effects were detected as detailed in Supplementary Figure 15. Other samples were not replicated. Micrographs in Figures 3 are representative of 6 of 9 egress events, from a total of 19 imaged midguts and 2 independent infections. Micrographs in Figure 4 are representative of 3 independent experiments.
Randomization	P. falciparum sporozoites were randomly assigned to control and activating conditions in figure 4. Randomization was not performed for other collections because of the exploratory nature of this work.
Blinding	Processing of samples downstream of cell sorting (cDNA, PCR, library prep and sequencing) was blinded. Cell collections and sorting were not blinded given the nature of different collection methods for the different stages.

Reporting for specific materials, systems and methods

We require information from authors about some types of materials, experimental systems and methods used in many studies. Here, indicate whether each material, system or method listed is relevant to your study. If you are not sure if a list item applies to your research, read the appropriate section before selecting a response.

Materials & experimental systems

n/a	Involved in the study
[ ]	[x] Antibodies
[x]	[ ] Eukaryotic cell lines
[x]	[ ] Palaeontology and archaeology
[x]	[ ] Animals and other organisms
[x]	[ ] Human research participants
[x]	[ ] Clinical data
[x]	[ ] Dual use research of concern

Methods

n/a	Involved in the study
[x]	[ ] ChIP-seq
[ ]	[x] Flow cytometry
[x]	[ ] MRI-based neuroimaging

Antibodies

Antibodies used

Antibodies against P. falciparum Pfs25 and CSP were used to purify ookinetes and sporozoites, respectively.

The reference for anti-Pfs25 antibody is the following: Delves, M. J. et al. Routine in vitro culture of P. falciparum gametocytes to evaluate novel transmission-blocking interventions. Nat. Protoc. 11, 1668–1680 (2016).

Anti-CSP was obtained through BEI Resources, NIAID, NIH: Monoclonal Anti-Plasmodium falciparum Circumsporozoite Protein (CSP), Clone 2A10 (produced in vitro), MRA-183A, contributed by Elizabeth Nardin."

Rabbit antibodies against MSP1-19 and PTEX150 were a gift from Paul Gilson, Burnett Institute.

Secondary antibodies (donkey anti-mouse AlexaFluor488 and donkey anti-rabbit AlexaFluor647) were from Jackson ImmunoResearch. Antibody dilutions are indicated in the methods section.

Validation

Pfs25 and CSP antibodies have been extensively used for immunolabeling of P. falciparum ookinete or sporozoite cells, however, we have validated their use for FACs purification of ookinetes and sporozoites as depicted in Supplementary Figures 1 and 3.

Antibodies against P. falciparum MSP1 and PTEX150 used in this study have been previously validated and shown to be specific:

anti-MSP1: Riglar, D., Rogers, K., Hanssen, E. et al. Spatial association with PTEX complexes defines regions for effector export into Plasmodium falciparum-infected erythrocytes. Nat Commun 4, 1415 (2013). https://doi.org/10.1038/ncomms2449

anti-PTEX150: de Koning-Ward, T. F., Gilson, P. R., Boddey, J. A., Rug, M., Smith, B. J., Papenfuss, A. T., Sanders, P. R., Lundie, R. J., Maier, A. G., Cowman, A. F., & Crabb, B. S. (2009). A newly discovered protein export machine in malaria parasites. Nature, 459 (7249), 945–949. https://doi.org/10.1038/nature08104

Flow Cytometry

Plots

Confirm that:

- [x] The axis labels state the marker and fluorochrome used (e.g. CD4-FITC).
- [x] The axis scales are clearly visible. Include numbers along axes only for bottom left plot of group (a 'group' is an analysis of identical markers).
- [x] All plots are contour plots with outliers or pseudocolor plots.
- [x] A numerical value for number of cells or percentage (with statistics) is provided.

Methodology

Sample preparation

Plasmodium cells from different stages of the parasite life cycle were labeled with antibodies specific for stage markers, DNA and RNA dyes, as explained in detail in Materials and Methods. Single cells were then sorted into 96-well plates.

Instrument

BD FACsAriaIII

Software

BD FACsDiva during acquisition and FlowJo for figure preparation.

Cell population abundance

The abundance of the cells sorted are shown in Figures S1-3.

Gating strategy

The gating strategies are depicted in great detail in Figures S1-3.

- [x] Tick this box to confirm that a figure exemplifying the gating strategy is provided in the Supplementary Information.
