# Supplementary material for: A single-cell atlas of Plasmodium falciparum transmission through the mosquito
Source: Nat Commun. 2021 May 27;12:3196. doi: 10.1038/s41467-021-23434-z (PMC8159942; doi:10.1038/s41467-021-23434-z)
Supplement: Supplementary file 2 — Peer Review File [file 41467_2021_23434_MOESM2_ESM.pdf]

Editorial Note: This manuscript has been previously reviewed at another journal that is not operating a transparent peer review scheme. This document only contains reviewer comments and rebuttal letters for versions considered at Nature Communications.

Reviewers' Comments:

Reviewer #1:
Remarks to the Author:
I feel that the authors have responded thoroughly to my previous comments and those of the other reviewers.

Reviewer #3:
Remarks to the Author:
The authors have adequately addressed all the concerns raised by myself and the other reviewers. In fact, I was surprised this manuscript was rejected after the initial submission given the relatively positive tone of the original reviews. I do not believe that the authors should be made to jump through any additional hoops.

Jim Collins

REVIEWERS' COMMENTS

Reviewer #1 (Remarks to the Author):

I feel that the authors have responded thoroughly to my previous comments and those of the other reviewers.

Reviewer #3 (Remarks to the Author):

The authors have adequately addressed all the concerns raised by myself and the other reviewers. In fact, I was surprised this manuscript was rejected after the initial submission given the relatively positive tone of the original reviews. I do not believe that the authors should be made to jump through any additional hoops.

Jim Collins

We thank both reviewers for the time they took to revise our manuscript and their positive feedback on the latest version.
